# Observed declines in upper ocean phosphate-to-nitrate availability

**DOI:** 10.1073/pnas.2411835122

**Published:** 2025-02-04

**Authors:** Skylar D. Gerace, Jun Yu, J. Keith Moore, Adam C. Martiny

**Affiliations:** ^a^Department of Earth System Science, University of California, Irvine 92697; ^b^Department of Ecology and Evolutionary Biology, University of California, Irvine 92697

**Keywords:** Redfield ratio, stoichiometry, nutrients, climate change, ocean

## Abstract

Climate warming is increasing ocean stratification, which in turn should decrease the nutrient flux to the upper ocean. This may slow marine primary productivity, causing cascading effects throughout food webs. However, observing changes in upper ocean nutrients is challenging because surface concentrations are often below detection limits. We show that the nutricline depth, where nutrient concentrations reach well-detected levels, is tied to productivity and upper ocean nutrient availability. Next, we quantify nutricline depths from a global database of observed vertical nitrate and phosphate profiles to assess contemporary trends in global nutrient availability (1972–2022). We find strong evidence that the P-nutricline (phosphacline) is mostly deepening, especially throughout the southern hemisphere, but the N-nutricline (nitracline) remains mostly stable. Earth System Model (ESM) simulations support the hypothesis that reduced iron stress and increased nitrogen fixation buffer the nitracline, but not phosphacline, against increasing stratification. These contemporary trends are expected to continue in the coming decades, leading to increasing phosphorus but not nitrogen stress for marine phytoplankton, with important ramifications for ocean biogeochemistry and food web dynamics.

## Significance

Earth System Models (ESMs) predict that ocean nutrients are decreasing from ocean warming with potentially severe impacts on marine life globally. However, direct observations have yet to support predictions due to the low detectability of nutrients throughout the ocean surface. Here, we quantified the depths where nitrate and phosphate reached well-detected concentrations through time over five decades. The temporal trends of these depths revealed that upper ocean phosphate is mostly declining, while nitrate is mostly stable. Using ESMs, we demonstrate that this difference is likely due to decreasing iron stress for nitrogen fixation, which replenishes nitrate with increasing stratification. These findings suggest that phosphorus limitation is expanding throughout the ocean, consequently bringing many important implications for marine ecosystems.

Nitrate and phosphate are key nutrients for ocean ecosystems (1), and Earth System Models (ESMs) predict that increasing ocean stratification due to climate warming is decreasing upper ocean nutrient supply (2–4). This trend can substantially reduce phytoplankton productivity and in turn negatively impact marine food webs (5). Consistent with these predictions, climate-induced declines of the nutrient supply coincide with reduced productivity throughout the geological record (6, 7). Despite these predictions, the expected decline in present-day surface nutrient availability has yet to be detected.

A major challenge with observing contemporary changes in nitrate and phosphate is that they are often below detection limits throughout the ocean surface (8, 9). However, both nutrients increase with depth (10). The nutricline depth, where either nutrient first reaches a well-detected concentration, commonly occurs below the surface where light limits photosynthesis and remineralized nutrients accumulate (11, 12). This depth has been proposed as a proxy for nutrient availability due to its relationships with phytoplankton productivity (13), resource demand (14), and community composition (15). Additionally, the nutricline depth is intimately linked with stratification, as a more stratified ocean typically exhibits weaker vertical mixing and reduced nutrient flux rates (16). Therefore, nutricline depths may proxy upper ocean nutrient availability at a global scale. Hence, we hypothesize that ongoing stratification has deepened nutricline depths worldwide in recent decades.

Using an ESM (CESM2-MARBL-8P4Z), we found support for the link between nutricline depths and upper ocean nutrient flux. We defined nutricline depths for nitrate (nitracline, Z_NO3_) and phosphate (phosphacline, Z_PO4_) based on well-measured threshold concentrations in Redfield proportions ([NO_3_^-^] = 3 µmol kg^−1^ and [PO_4_^3−^] = 3/16 µmol kg^−1^, *Materials and Methods*) (17). Nutricline depths showed strong negative correlations with nutrient fluxes (*SI Appendix*, Fig. S1 *A* and B). They also negatively correlated with organic carbon export (*SI Appendix*, Fig. S1 *C* and D), demonstrating their link to ecosystem processes such as carbon sequestration. Additionally, our selected thresholds yielded some of the strongest correlations and lowest prediction errors between nutrient flux and nutricline depths (*SI Appendix*, Fig. S2). These model validations indicate that nutricline depths defined from our selected thresholds effectively represent nutrient and broader ecosystem dynamics across the global ocean.

**Fig. 1.**
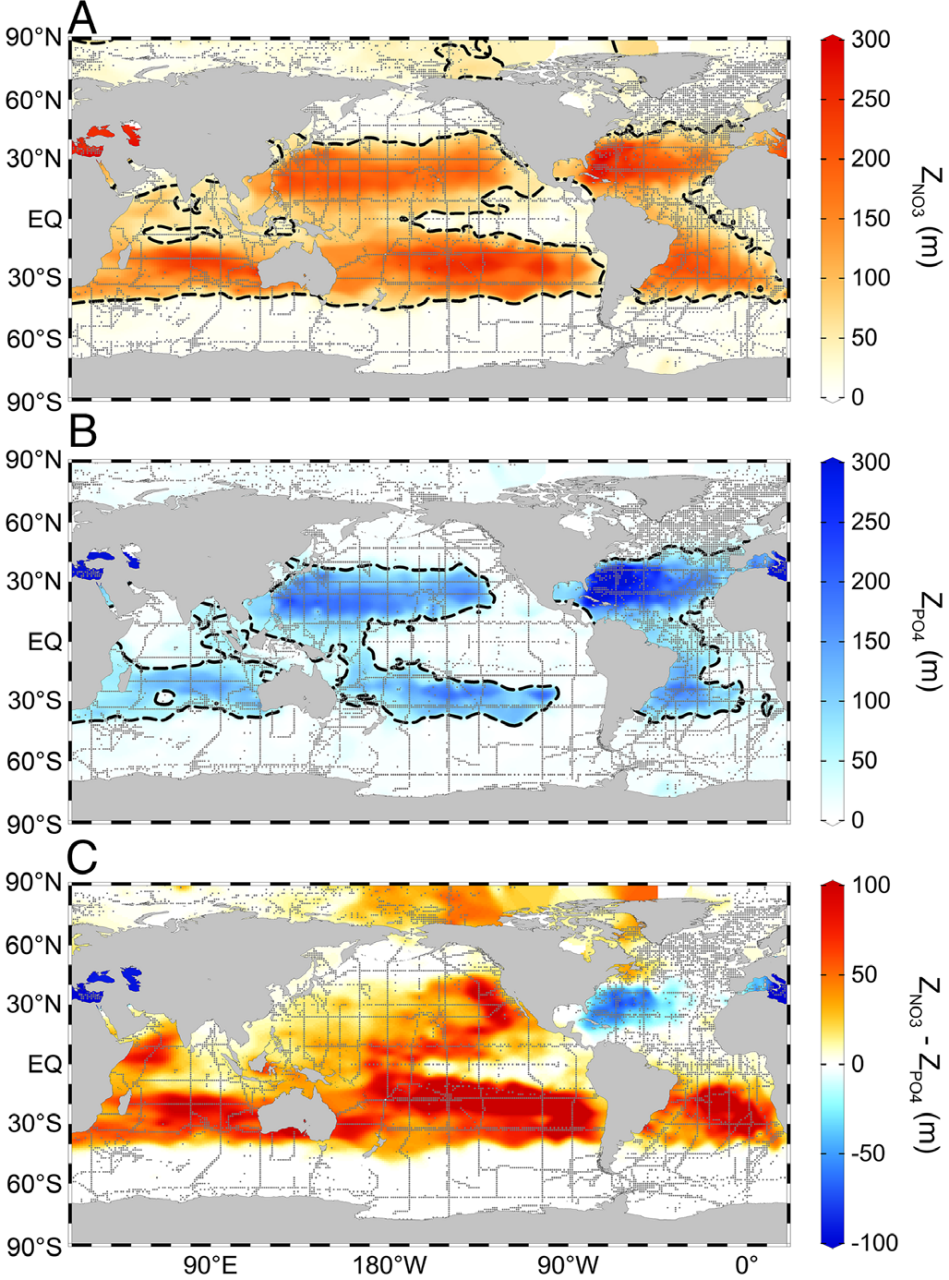
Mean nutricline depths were consistent with patterns of nutrient availability and limitation. This figure depicts nutricline depths from GO-SHIP. (*A*) Nitracline depths (Z_NO3_) and (*B*) phosphacline depths (Z_PO4_) depicted here are average values for all of 1972–2022 at each unique pair of geographic coordinates (gray dots; sites for Z_NO3_, n = 8,508; sites for Z_PO4_, n = 9,033). The thresholds for defining these nutricline depths were 3 µmol kg^−1^ and 3/16 µmol kg^−1^ for nitrate and phosphate, respectively. The dashed line is the contour of 50 m depth; we determined trends from nutriclines deeper than 50 m. (*C*) The difference of average nitracline and phosphacline depths (sites for Z_NO3_−Z_PO4_, n = 8,284). These maps show values that were spatially interpolated using DIVA in Ocean Data View.

With model simulations supporting nutriclines as proxies for nutrient supply, we proceeded to quantify nutricline depths from cruise observations spanning the past five decades (1972–2022). Our core analysis included 31,715 nitrate and 37,549 phosphate depth profiles measured using standardized analytical techniques by the Global Ocean Ship-based Hydrographic Investigations Program (GO-SHIP). Spatially, mean nitracline and phosphacline depths are deepest in areas with the lowest surface concentrations and when high nutrient stress was detected by bioassay experiments and genetic biomarkers (18–20) (Fig. 1 and *SI Appendix*, Fig. S3). Deep nutriclines are predominantly found at low and middle latitudes (Fig. 1 *A* and *B*), in agreement with the notion that oligotrophic waters are mostly within tropical and subtropical regions (21). The phosphacline is only deeper than the nitracline in the North Atlantic subtropical gyre and Mediterranean Sea (Fig. 1*C*), which are known P-stressed regions (20, 22, 23). There is also an east-west phosphacline vs. nitracline gradient in the North Pacific Ocean matching the spatial variance of sparse surface phosphate concentrations measured with high sensitivity techniques (9). The nitracline is much deeper than the phosphacline in each of the southern subtropical gyres (Fig. 1*C*). We found that nutricline depths from two larger, but more heterogeneous datasets [Global Ocean Data Analysis Project (GLODAP) and World Ocean Database (WOD)], have nearly identical spatial distributions to the depths obtained using data from GO-SHIP (*SI Appendix*, Fig. S4). In summary, model predictions and global observations both suggest that nutricline depths can reliably proxy upper ocean nutrient dynamics.

## Nutricline Trends Revealed Declining Phosphate-to-Nitrate Availability

To test our hypothesis of a deepening nutricline, we fitted two separate linear regressions to global nitracline and phosphacline depths (*SI Appendix*, Fig. S5). The nitracline regression shows no significant change over time (*P* = 0.07). However, the phosphacline significantly deepened (*P* = 9 × 10^−7^) by 23 m total over the past five decades (Z_PO4_ slope = 0.47 m y^−1^, slope SE = 0.10 m y^−1^) (*SI Appendix*, Fig. S5 *A* and B and Table S1). To put this rate into perspective, the average phosphacline depth in southern oligotrophic waters (45°S to 1°S) is only 62 m, and the average difference between the nitracline and phosphacline depths (Z_NO3_–Z_PO4_) is only 31 m throughout the global oligotrophic ocean (45°S to 45°N) (*SI Appendix*, Table S2). To account for seasonality in sampling, we corrected observed nutricline depths by subtracting their respective monthly climatological value. Again, the phosphacline exhibits a deepening trend, whereas the nitracline displays no temporal trend (*SI Appendix*, Fig. S5 *C*–E). However, using different global datasets or threshold concentrations does sometimes indicate nitracline deepening, although this deepening rate never exceeds the rate for phosphacline depths (*SI Appendix*, Table S1). Also, nitracline and phosphacline slopes were not significantly different for the lowest threshold concentrations, likely due to measurement uncertainty and associated poorly resolved phosphacline depths at these low concentrations. Nonetheless, these global regressions consistently show that the phosphacline is, on average, deepening faster than the nitracline.

**Fig. 2.**
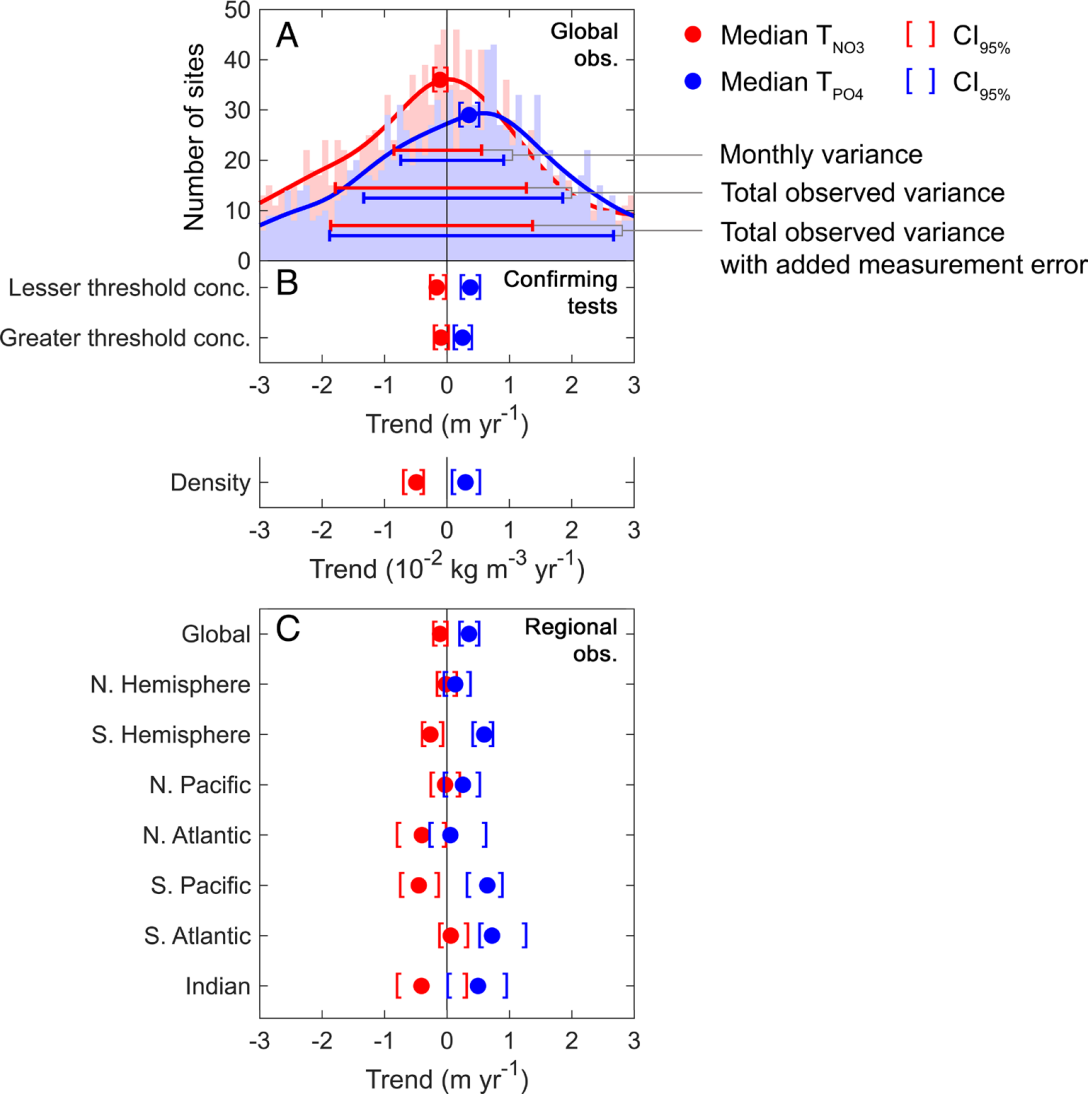
Nutricline trends revealed declining phosphate-to-nitrate supply worldwide. Nutricline depths were defined based on threshold concentrations of [NO_3_^−^] = 3 μmol kg^−1^ and [PO_4_^3−^] = 3/16 μmol kg^−1^. (*A*) Site-specific trends for each unique pair of geographic coordinates (sites for T_NO3_, n = 1,859; sites for T_PO4_, n = 1,641). 95% confidence intervals (CI_95%_) were calculated for each median trend by generating 10,000 bootstrap samples of the respective dataset. The curves over the histograms depict the kernel densities. The sets of error bars from top to bottom are the interquartile ranges of T_NO3_ and T_PO4_ from the WOA18 monthly climatology, GO-SHIP observations, and GO-SHIP observations with added measurement error. (*B*) Medians of T_NO3_ and T_PO4_ when defining nutricline depths based on lesser ([NO_3_^−^] = 1 μmol kg^−1^ and [PO_4_^3−^] = 1/16 μmol kg^−1^) and greater ([NO_3_^−^] = 5 μmol kg^−1^ and [PO_4_^3−^] = 5/16 μmol kg^−1^) threshold concentrations, additionally, the global density trends of nutricline depths. *SI Appendix*, Table S3 for detailed statistics of these observed trends. (*C*) Regional medians of T_NO3_ and T_PO4_. *SI Appendix*, Table S4 for detailed descriptions of these regions and their respective statistics.

We next quantified site-specific temporal trends for nitracline (T_NO3_) and phosphacline (T_PO4_) depths (Fig. 2 and *SI Appendix*, Table S3). This was done to eliminate the possibility that the spatiotemporal variability in sampling induced a spurious trend; for example, by overrepresenting locations with particularly deep nutriclines at specific years. The median T_NO3_ is negative [−0.11 m y^−1^, CI_95%_ = (−0.22, −0.02)] indicating a possible shoaling. In contrast, the median T_PO4_ shows a phosphacline deepening [0.35 m y^−1^, CI_95%_ = (0.20, 0.49)]. Similar to the global regression, this suggests a general deepening of the phosphacline at a rate of 18 m over the past 50 y. Thus, the site-specific trends show that only the phosphacline is mostly deepening, whereas the nitracline is mostly stable or could even be shoaling.

We also observed extensive variability among site-specific trends (Fig. 2*A*), including both deepening and shoaling nutriclines across ocean regions. The timing for each sample and associated section cruise is not controlled. Therefore, the trend for any site will be sensitive to a range of mechanisms, including seasonality, that contribute to shifts in the nutricline. However, we will next show that none of the tested mechanisms can account for the long-term phosphacline deepening. Simulating the actual sampling dates onto a fixed monthly climatology (WOA18 with no long-term change) resulted in interquartile ranges of T_NO3_ and T_PO4_ that are about 50% of the observed variances. This suggests that monthly variability in nutriclines is an important source of variation in site-specific trends (Fig. 2*A* and *SI Appendix*, Fig. S6 *C* and D). Next, we constructed 10,000 random populations of the fixed monthly climatology to robustly quantify the effect of seasonal variance without any long-term changes (*SI Appendix*, Fig. S6 *E* and F). The median trends in nutricline depths are indistinguishable from zero, showing that monthly variability does not lead to an overall bias in median trends. We then introduced an artificial fixed long-term trend at all sites in the random populations. This resulted in the same variance with different locations showing a mix of deepening or shoaling trends in agreement to what we found in the actual observations. However, the median shifted to match the fixed imposed trend (*SI Appendix*, Fig. S6 *G* and H). We interpret these analyses as the observed median capturing the actual underlying tendency in trends, whereas the variance in site-specific trends is mostly attributed to seasonality. To match both the observed median trends and overall variance in depths, each nitracline trend had to be sampled from a normal distribution with a shoaling tendency (mean = −0.63 m y^−1^, SD = 1.5 m y^−1^), and each phosphacline trend came from one with a deepening tendency (mean = 0.20 m y^−1^, SD = 1.0 m y^−1^) (*SI Appendix*, Fig. S6 *I* and J). This demonstrates that the median trend robustly captures the underlying tendency of the site-specific trends despite their high variability. In summary, these analyses show that sampling timing contributes to the observed variability in trends between sites, but any overall shift in the median must come from a long-term change in nutricline depths.

Outside of seasonality, variability in the site-specific trends could also arise from measurement error or interannual climate forcings. When we simulated measurement uncertainty to the observed nutrient concentrations, we found that the interquartile range of T_NO3_ only increased about 5% (Fig. 2*A*). In contrast, the interquartile range of T_PO4_ increased about 40% due to the lower threshold concentration (Fig. 2*A*). This suggests that measurement error is a considerable factor for the variability of T_PO4_ but not T_NO3_, despite both having nearly equal total variance. However, accounting for sampling error to nutrient concentrations still yielded a median T_NO3_ indicative of shoaling, and a median T_PO4_ indicative of deepening [median T_NO3_ = −0.18 m y^−1^, CI_95%_ = (−0.27, −0.03); median T_PO4_ = 0.26 m y^−1^, CI_95%_ = (0.09, 0.43)]. Part of the total variance could also be attributed to ENSO or other climate modes, which can affect upper ocean nutrient profiles on interannual timescales (24). We found that during some El Niño years, the global median nitracline and phosphacline depths were relatively deeper (*SI Appendix*, Fig. S7). Thus, the combination of seasonal and interannual changes in nutrient concentrations together with measurement uncertainty explains most of the observed variance in nutricline trends.

Despite a large variability, there is strong evidence that the median values of T_NO3_ and T_PO4_ are both significant and capture long-term changes. To confirm their significance, we scrambled the sampling time to construct 10,000 randomized conglomerate datasets and found the median trends for each of these random populations (MToRP). This showed that the probabilities of the observed medians in T_NO3_ and T_PO4_ being zero are less than 2 × 10^−2^ and 1 × 10^−5^, respectively when compared to 10,000 randomized datasets (*SI Appendix*, Table S3). Moreover, sign and Kruskal–Wallis tests also supported that both median trends are significantly different from zero and each other (*SI Appendix*, Table S3). To assess whether the medians captured long-term changes, we referred to nutricline depths from two high-resolution time-series, the Bermuda Atlantic Time-series Study (BATS), and A Long-term Oligotrophic Habitat Assessment (ALOHA) (25). Despite notable short-term variability in nutricline depths, the time-series data suggest that both hemispheres and all five subtropical gyres had sufficient sample sizes for their median trends to reflect long-term changes (*SI Appendix*, Fig. S8 and *Materials and Methods* in *SI Appendix*). Together, these findings robustly demonstrate that the global and regional declines in phosphate availability withstand extensive statistical validation.

The median trends are also consistent across different observational products and choice of threshold concentrations (Fig. 2*B* and *SI Appendix*, Table S3). WOD hosts nearly an order of magnitude more nutrient observations than GO-SHIP, but these observations were collected with a diversity of protocols. Nonetheless, WOD similarly shows overall shoaling and deepening trends of the nitracline and phosphacline, respectively (*SI Appendix*, Table S3). The GLODAPv2.2022 adjusted product also shows that any measurement bias with time has little influence on the median trends (*SI Appendix*, Table S3). Additionally, we defined nutricline depths using different concentrations and N:P proportions for thresholds and found similar medians for site-specific trends through time (*SI Appendix*, Table S3). Therefore, the median trends across different databases and definitions agree that phosphacline depths are mostly deepening, whereas nitracline depths are constant or shoaling (Fig. 2*B* and *SI Appendix*, Table S3).

We finally explored the robustness of the median trends with a variety of additional tests. First, we omitted extreme values of T_NO3_ and T_PO4_ based on what the time-series data suggest are feasible (*SI Appendix*, Fig. S8 *A*–D) and still detected phosphacline deepening (*Materials and Methods* in *SI Appendix*). Second, the phosphacline deepening is robust after filtering out more than 90% of cruise observations based on sampling density (*SI Appendix*, Fig. S9 and *Materials and Methods* in *SI Appendix*). Third, we found a negative median trend for the paired residual Z_NO3_ − Z_PO4_ [−0.36 m y^−1^, CI_95%_ = (−0.46, −0.27)], which also suggests a faster deepening of the phosphacline relative to the nitracline (*SI Appendix*, Fig. S10). Fourth, there is no indication of a bias in nutrient concentrations due to changing measurement methods during the observation period. After subtracting observed and mean climatological concentrations, we did not find any negative trends at depths well below the nutricline where concentrations presumably should be stable (*SI Appendix*, Fig. S11). We did detect negative trends in the upper ocean but only for phosphate concentrations, which further supports that we found changes in phosphate availability and not just phosphacline depths. Finally, the water density at the depth of the nitracline and phosphacline show decreasing and increasing trends, respectively [median T_NO3_ = −5 × 10^−3^ kg m^−3^ y^−1^, CI_95%_ = (−7 × 10^−3^, −4 × 10^−3^); median T_PO4_ = 3 × 10^−3^ kg m^−3^ y^−1^, CI_95%_ = (8 × 10^−4^, 5 × 10^−3^); Fig. 2*B*]. This indicates that the nutricline depths are shifting independently of density layers, suggesting that biological processes may control the nutricline depth trends.

Based on the combination of these tests and analyses, we conclude that there is strong support for a general deepening of the phosphacline. It is less certain whether the nitracline is stable or possibly shoaling, but it is clearly not deepening as fast as the phosphacline. Thus, our analysis points to differential shifts in nutricline depths for these two nutrients, revealing a contemporary depletion of upper ocean phosphate relative to nitrate.

## A Hemispherical Difference in Nutricline Trends

A regional analysis supports a significant hemispheric difference in the observed nutricline trends (Fig. 2*C* and *SI Appendix*, Table S4). Neither nutricline depths show any significant trends in the northern hemisphere. If we split the northern hemisphere into basins, the median T_NO3_ is significantly negative (shoaling) in the North Atlantic, whereas the median T_PO4_ is marginally positive (deepening) in the North Pacific. Across the southern hemisphere, the median trends are indicative of phosphacline deepening and nitracline shoaling, although the medians are not significant for the Indian Ocean (Fig. 2*C* and *SI Appendix*, Table S4). In general, the observed nitracline shoaling and phosphacline deepening are much stronger in southern [median T_NO3_ = −0.27 m y^−1^, CI_95%_ = (−0.40, −0.09); median T_PO4_ = 0.60 m y^−1^, CI_95%_ = (0.41, 0.71)] compared to northern basins [median T_NO3_ = −0.02 m y^−1^, CI_95%_ = (−0.16, 0.13); median T_PO4_ = 0.13 m y^−1^, CI_95%_ = (−0.05, 0.35)] (*SI Appendix*, Table S4). Thus, the decline in upper ocean phosphate-to-nitrate availability is most pronounced in the southern hemisphere.

## Possible Biogeochemical Mechanisms for the Observed Trends

There are multiple possible biogeochemical drivers for shifting nutricline depths, including changes in phytoplankton resource demands, external nutrient inputs (riverine or atmospheric), or biological nitrogen fixation. First, it is commonly proposed that phytoplankton have an increasing N:P demand in a warming ocean due to a reduction of P-rich ribosomes needed for protein synthesis (26). We find that this biochemical mechanism is unlikely to explain our results as it implies a faster deepening of the nitracline relative to the phosphacline. Second, changes in riverine inputs (27) and atmospheric deposition (28) of predominantly nitrogenous material can increase nitrate and the drawdown of phosphorus relative to nitrogen. This mechanism is also unlikely to explain our results, as increasing nitrogenous input is primarily occurring in the northern hemisphere (27, 28), whereas a deepening phosphacline is mostly observed in the southern hemisphere (Fig. 2*C* and *SI Appendix*, Table S4). Hence, neither changes in phytoplankton demands nor external inputs relate well to the observed nutricline trends.

A third possibility is that changes in biological nitrogen fixation compensate for a declining supply of nitrate from stratification, but not for phosphate (23). Marine nitrogen fixation is often limited by iron (29–31), and for many reasons, this iron stress may be decreasing (32). Jiang et al. found that *Trichodesmium* cells use iron more efficiently at elevated temperature (33). Moreover, increasing stratification results in an increased Fe:N supply ratio by moderating the relative importance of vertical vs. aeolian nutrient supply (34). Deposition of soluble iron from combustion sources, including wildfire and fossil fuel burning, has been increasing in recent decades (35), and significantly impacting marine biogeochemistry (36). Considering these findings, diazotrophs are more commonly limited by iron in the southern and phosphorus in the northern hemisphere (37). Decreasing iron stress will therefore mostly stimulate nitrogen fixation and phosphate drawdown in the southern hemisphere. Hence, we hypothesize that the regulation of nitrogen fixation is important for the observed decline in phosphate-to-nitrate availability and can explain the hemispherical difference in depth trends.

## Nutricline Trends Relate with Ocean Nitrogen Fixation and Stratification in CMIP6

**Fig. 3.**
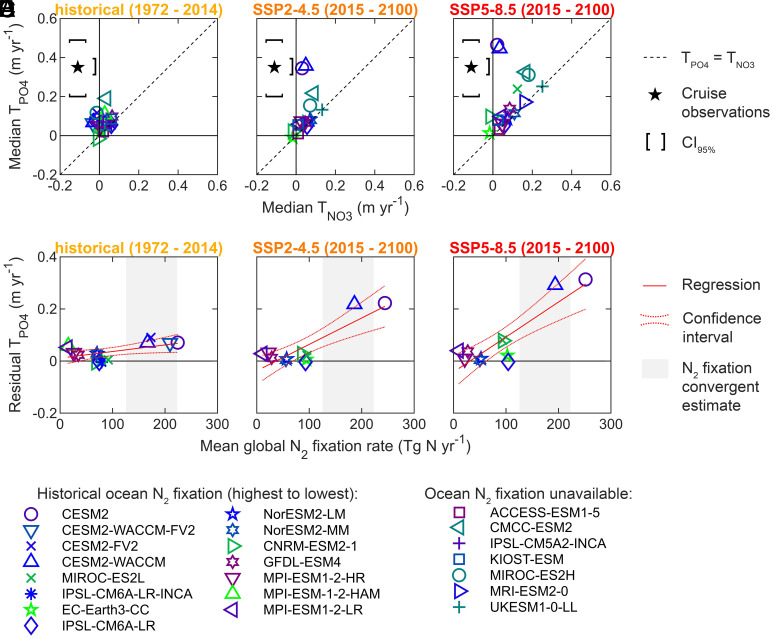
CMIP6 nutricline trends were linked to ocean nitrogen fixation. The first row shows CMIP6 predicted T_NO3_ and T_PO4_ under (*A*) historical, (*B*) middle-of-the-road (SSP2-4.5), and (*C*) business-as-usual (SSP5-8.5) emission scenarios (i.e., different stratification scenarios). (*D*–*F*) The second row is the relationship between declining phosphate-to-nitrate availability and ocean nitrogen fixation under each three scenarios. The decline of phosphate-to-nitrate availability was quantified as the residual T_PO4_, which was the orthogonal distance from the T_PO4_ = T_NO3_ line. The CMIP6 nitrogen fixation rates were global estimates averaged for the entire scenario time period. The convergent estimate of global ocean nitrogen fixation was quantified by Wang et al. using an inverse model and an Earth System Model (ESM) (37).

To test this hypothesis, we compared trends in nitracline and phosphacline depths in the CMIP6 ESMs across historical and future emission scenarios (i.e., less and more stratified scenarios, respectively) (Fig. 3 *A*–*C*). In the historical scenario, all models predicted nutricline trends notably smaller than the observed trends. In some models, equivalent trends for the nitracline and phosphacline depths at Redfield proportions (i.e., T_PO4_ = T_NO3_) were seen. For other models, the phosphacline showed a faster deepening trend compared to the nitracline. The opposite was never seen. These findings generally held true under future emission scenarios, although most models predicted parallel faster deepenings of both nitracline and phosphacline depths. In conclusion, greater stratification generally led to a faster nutricline deepening in CMIP6, but some models included biogeochemical processes causing a faster deepening of the phosphacline relative to the nitracline.

The faster deepening of the phosphacline relative to the nitracline was correlated with ocean nitrogen fixation rates across ESMs (Fig. 3 *D*–*F*). A differential deepening (i.e., residual T_PO4_) was quantified as the orthogonal distance from the T_PO4_ = T_NO3_ line. Across all three model scenarios, we found significant positive relationships between ocean nitrogen fixation rates and the residual T_PO4_ (*SI Appendix*, Table S5). The correlation coefficients and regression slopes also increased with stronger emissions. This shows that the relationship between nitrogen fixation and declining phosphate-to-nitrate availability is dependent on the rate of ocean warming and increasing stratification. Therefore, the CMIP6 model dynamics were consistent with the hypothesis that nitrogen fixation with stratification determines the faster deepening of the phosphacline relative to the nitracline.

## Nutricline Depths are Influenced by Nutrient Uptake Rates and Iron Deposition in CESM2

Among the CMIP6 models, CESM2 had the highest ocean nitrogen fixation rates as well as the highest residual T_PO4_. Although higher than most, this model was consistent with the convergent estimate of ocean nitrogen fixation quantified by Wang et al. (37). Also, CESM2 showed nutricline trends that were the most consistent with observations. Hence, we next investigated different processes in CESM2 affecting nitracline and phosphacline trends.

Flexible phytoplankton biomass composition may slow the deepening of the phosphacline relative to the nitracline. With a CESM2 model, Kwon et al. found that flexible rather than Redfield phosphate requirement led to reduced upper ocean phosphate demand by 2100 (38). We analyzed these same experiments and found that flexible phosphate uptake also led to shallower phosphacline depths (*SI Appendix*, Fig. S12 *A*–D) (38). However, flexible phosphate uptake did not have a major effect on nutricline trends through time (*SI Appendix*, Fig. S12*E*). Therefore, flexible nutrient uptake stoichiometry impacted the initial depths of nutriclines but was not a factor for the long-term trends.

**Fig. 4.**
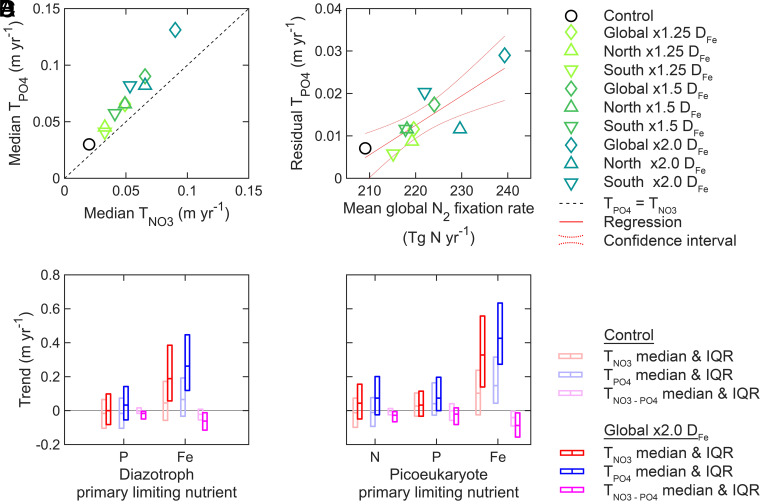
Increasing iron deposition in CESM2 augmented nitrogen fixation and phosphate-to-nitrate depletion. (*A* and *B*) The first row shows median nutricline trends from each iron deposition (D_Fe_) experiment and the relationship between the residual T_PO4_ (i.e., the orthogonal distance from the T_PO4_ = T_NO3_ line) and ocean nitrogen fixation rate. (*C* and *D*) The second row shows nutricline trends at sites grouped by primary nutrient limitation at the run’s starting year. The center of each rectangle is the median trend, and the top and bottom edges are the 25th and 75th percentiles. T_NO3-PO4_ is the trend of Z_NO3_–Z_PO4_.

Decreasing iron stress may enhance the deepening of the phosphacline relative to the nitracline through stimulation of nitrogen fixation (34, 35). In CESM2, we adjusted iron stress by increasing atmospheric iron deposition (D_Fe_) rates either by hemisphere or globally (Fig. 4). Increased deposition rates increased nitrogen fixation and resulted in a faster deepening of the phosphacline relative to the nitracline (Fig. 4 *A* and *B*). We again found a significant relationship between the residual T_PO4_ and integrated nitrogen fixation [slope = 7 × 10^−4^ m × (Tg N_fix_)^−1^, slope SE = 2 × 10^−4^ m × (Tg N_fix_)^−1^, *P* = 3 × 10^−3^, n = 10]. Additionally, we determined nutricline trends in regions with different primary nutrient limitations (Fig. 4 *C* and *D*). Increased D_Fe_ led to a faster deepening of the phosphacline relative to the nitracline no matter the location, but the strongest effect was in iron-limited regions. This suggested that as iron stress was alleviated, it was eventually replaced with increased phosphorus stress. Thus, an increase in nitrogen fixation by alleviating iron stress directly results in a faster deepening of the phosphacline relative to the nitracline.

## Caveats and Conclusions

We present evidence for a deepening of the phosphacline that is decoupled from any commensurate change in the nitracline. However, there are caveats to be considered. First, we restricted our global trend analysis to nutricline depths that were deeper than 50 m and from 45°S to 45°N. Omitting depths shallower than 50 m did not appreciably affect the observed trends, as they remained consistent when including depths deeper than 25 m (*SI Appendix*, Tables S1 and S3). The applied latitudinal range removed the Arctic Ocean from our analyses, despite this region experiencing some degree of nitrogen limitation (39). However, the sampling density in the Arctic was too low to quantify trends from nutricline depths that met our criteria. Furthermore, the Arctic experiences high variability in sea-ice cover, which is a major driver of stratification absent from the rest of the oligotrophic ocean (40). Second, there were notably less cruise observations from the earliest and latest years, but we still found a significantly faster deepening of the phosphacline from 1982 to 2012 (*SI Appendix*, Tables S1 and S3). Third, ocean basins were sampled at different frequencies leading to less statistical power for some basins, most notably for the Indian Ocean. However, the nitrate and phosphate depth profiles were mostly sampled concurrently throughout time and space; thus, the sampling variability is not expected to have any effect on the differences in the trends observed for the two nutrients (*SI Appendix*, Fig. S13). Fourth, although the global trends were robust, we recognize that variability in the phosphacline trends were more influenced by measurement error than the nitracline trends. Accounting for these caveats, we still find significant evidence for declining phosphate-to-nitrate availability over the past five decades (1972–2022).

The cruise observations suggest that phosphacline depths deepened by approximately 20 m integrated over the past five decades, a rate that is almost an order of magnitude larger than most CMIP6 predictions for the historical period (Figs. 2 and 3 and *SI Appendix*, Tables S1 and S3). We hypothesize that CMIP6 underestimated the increase of iron inputs over the historical period (35), as many models utilize climatological iron deposition that would not capture any trends (41, 42). At the observed rate, nutrient availability throughout the southern oligotrophic gyres may soon resemble the western side of the North Pacific subtropical gyre (Fig. 1*C* and *SI Appendix*, Table S2). This transition is possible due to the predicted decrease in iron stress for nitrogen fixation in this region (34, 35, 43). In turn, nutrient availability in the western North Pacific subtropical gyre may soon resemble the North Atlantic subtropical gyre with widespread phosphorus stress (Fig. 1*C* and *SI Appendix*, Table S2). A trend toward more phosphorus stress possibly driven by nitrogen fixation has already been observed in this area (44, 45). Therefore, the current patterns and trends in nutricline depths provide a basis for predicting which regions will begin to show increasing phosphorus stress for marine productivity.

Increasing phosphorus stress applies a selective pressure on marine phytoplankton, but some implications for ecosystems and elemental cycles remain uncertain. For instance, there is conflicting evidence whether total productivity should decline solely due to phosphorus limitation. Although phosphate has been suggested to be the ultimate control on phytoplankton production (1), variability in phytoplankton C:P and nitrogen fixation may buffer productivity despite increasing stratification (38, 46). In contrast, increasing nitrogen stress might have greater impacts on marine productivity, as phytoplankton have less ability to acclimate by modifying their C:N ratio (47). Nonetheless, phosphorus limitation does select for higher C:P in phytoplankton (48, 49), consequently reducing food quality throughout many marine food webs (50). These potential changes for marine ecosystems underscore the importance of constraining the rate of rising phosphorus stress throughout the ocean.

Increasing stratification and atmospheric iron inputs will likely continue in the coming decades (3, 32), but the decline in phosphate-to-nitrate availability is still subject to change. A declining phosphate supply would eventually start to limit nitrogen fixation rather than iron (51). Furthermore, continued deoxygenation from warming also increases denitrification and anammox, leading to greater declines in nitrate supplies (52). Therefore, the global trend toward phosphorus limitation could be temporary, but with an unknown duration. Continued efforts to monitor multiple nutrients and improve their model predictions should increase understanding of upper ocean biogeochemistry as the ocean continues to warm and stratify.

## Materials and Methods

Here, we describe the major components of our methodology, but a more detailed description is included in Supporting Information. We quantified nitracline and phosphacline depths (Z_NO3_ and Z_PO4_, respectively) worldwide from 1972 to 2022 using multiple conglomerate datasets of cruise observations [GO-SHIP (53), GLODAPv2.2022 (54), and WOD (55)]. For each cruise cast, we defined nitracline and phosphacline depths as the depths where interpolated nitrate and phosphate concentrations reached well-measured threshold concentrations. Our analyses mostly focus on results from threshold concentrations that were in Redfield proportions (e.g., [NO_3_^−^] = 3 µmol kg^−1^ and [PO_4_^3−^] = 3/16 µmol kg^−1^) (17). The geographic coordinates of each cast were rounded to the nearest degree, and we recorded the annually averaged depths for every unique pair of rounded coordinates, or site.

We determined trends through time using only annually averaged depths that were deeper than 50 m and from 45°S to 45°N. This was to ensure that the trends capture changes in nutrient-limiting regions and to mitigate effects from mixed layer variability (56). We first applied two separate linear regressions to the annually averaged nitracline and phosphacline depths through time. Next, for each unique site with annually averaged depths for two or more years, we fitted a linear regression to determine the site-specific trend (T_NO3_ and T_PO4_). We determined the 95% confidence intervals for each global median of the site-specific trends by generating 10,000 bootstrap samples of the respective dataset.

We tested the robustness of the observed depth trends with a series of confirmational analyses. First, we quantified nutricline depths in the WOA18 monthly climatology (57) using a similar methodology as the cruise observations to analyze temporal variability across sites. Second, we simulated the effects of additional measurement error by randomly adding a value to each observed nitrate and phosphate concentration. We selected values for nitrate from a normal distribution centered at zero with a standard deviation of 800 nmol kg^−1^, and values for phosphate from another distribution with a standard deviation of 100 nmol kg^−1^ (58). Third, we constructed 10,000 randomizations of each conglomerate cruise dataset by scrambling the years that nutricline depths were sampled. We compared the median site-specific trends from observations to the distribution of median trends from the 10,000 random populations (MToRP). Fourth, we subtracted interpolated nutrient concentrations from the observations and another monthly climatology [GLODAPv2.2016 (59)] to test whether nutrient detectability improved with time (i.e., a negative trend for concentrations below 1000 m depth).

We quantified nutricline depths and site-specific trends from different ESMs (60) using a similar methodology as the cruise observations. We examined the relationships between nutricline depths and nutrient flux using an updated version of CESM2-MARBL, which includes 8 types of phytoplankton and 4 types of zooplankton with different biogeochemical pathways (CESM2-MARBL-8P4Z) (61, 62). We also determined the effect of variable atmospheric iron deposition on nutricline trends using CEMS2-MARBL-8P4Z. We determined the effect of variable phosphate uptake on trends by quantifying nutricline depths from fixed and variable phytoplankton C:P renditions of CESM2 by Kwon et al. (38).

## Supplementary Material

Appendix 01 (PDF)

## Data Availability

In situ nutrient data have been deposited in https://cchdo.ucsd.edu/ (53), and nutricline data from the cruise observations are provided as Dataset S1. Dataset S1 and all of the essential code for analyzing the observations and models are deposited in Dryad, https://doi.org/10.5061/dryad.v41ns1s4v (63). Interpolated maps were created using Ocean Data View, and all statistical analyses were performed using MATLAB R2021b.
