# Supplementary material for: Observed declines in upper ocean phosphate-to-nitrate availability
Source: Proc Natl Acad Sci U S A. 2025 Feb 4;122(6):e2411835122. doi: 10.1073/pnas.2411835122 (PMC11831131; doi:10.1073/pnas.2411835122)
Supplement: Supplementary file 1 — Appendix 01 (PDF) [file pnas.2411835122.sapp.pdf]

PNAS

Supporting Information for

Observed declines in upper ocean phosphate-to-nitrate availability

Skylar D. Gerace, Jun Yu, J. Keith Moore, Adam C. Martiny*

*Corresponding author: amartiny@uci.edu

The PDF file includes:

Materials and Methods
Figs. S1 to S13
Tables S1 to S6
SI References (*1 – 41*)

Other supporting materials for this manuscript include the following:

Dataset S1

Materials and Methods:

Conglomerate cruise data sets

We quantified nutricline depths from 1,060 cruise data sets provided by the CLIVAR and Carbon Hydrographic Data Office (CCHDO) (*1*). All data was formatted in a standardized netCDF4 format following the Climate and Forecast (CF) Conventions version 1.8. These data sets included hydrographic data measured mostly as part of the Global Ocean Ship-Based Hydrographic Investigations Program (GO-SHIP). Other precursor programs of repeat hydrography, such as the World Ocean Circulation Experiment (WOCE), were included as well. We refer to the conglomerate data set from CCHDO as the GO-SHIP data set throughout the main manuscript.

We also compared our results from GO-SHIP with results from two other conglomerate data sets. We defined nutricline depths from 1,086 cruise data sets provided by the Global Ocean Data Analysis Project merged and adjusted data product (GLODAPv2.2022) (*2, 3*). Nutrient concentrations in GLODAPv2.2022 were adjusted to account for measurement biases with time (*2, 3*). Therefore, GLODAPv2.2022 may have revealed potential measurement biases in the GO-SHIP data set. We also referred to nutrient concentrations found in the World Ocean Database (WOD) (*4*). Although WOD did not prioritize repeat hydrography, this database held 12,707 unique cruise data sets from 1972 to 2022. Despite having the least amount of cruise data sets, we chose to focus our analysis on the GO-SHIP data set since its purpose is to provide consistent, quality-controlled measurements of repeat hydrography.

Cruise sampling methods

On the GO-SHIP cruises, temperature and salinity were recorded at various depths and stations using a Conductivity Temperature Depth (CTD) instrument (*5*). Nutrients were sampled from seawater collected by a Niskin bottle rosette fitted (*6*). Nutrient concentrations were photometrically determined from the collected seawater via continuous flow analysis. Nitrate was determined by first reducing nitrate to nitrite in a copperized cadmium column. A sulfanilamide solution and N-Napthylethylene-diamine were introduced to produce a red azo dye (*6*, *7*). To determine phosphate, molybdic acid was introduced to form phosphomolybdic acid. The phosphomolybdic acid was reduced to phosphomolybdous acid with hydrazine to produce a blue solution (*6*, *7*).

Nutrient concentrations

Here, we analyzed each cast where nitrate or phosphate was measured. Nitrate profiles inclusive of nitrite concentrations (i.e., nitrate + nitrite) were excluded from our analysis, as were any negative or outlier concentrations. We defined outlier concentrations as instances where a nutrient decreased with depth by more than 10 µmol kg^{-1} in the upper ocean. Nutrient concentrations were predominantly in units of µmol kg^{-1}, but for concentrations expressed as µmol L^{-1}, we converted them to µmol kg^{-1} by calculating seawater density. We calculated density using a UNESCO formula by incorporating temperature (°C) and salinity (psu) measurements included in each bottle file (*8*).

Interpolating nutrients with pressure

For every cast with nutrient measurements at 2 or more pressures, we linearly interpolated nitrate and phosphate concentrations for every 1 dbar down to a maximum pressure of 1000 dbar. The interpolation only spanned between the lowest and highest pressures with nutrient

measurements. Similarly, we interpolated temperature and salinity from the respective cruise CTD file for every 1 dbar. We then recorded the nutricline as the pressure and density where the interpolated nutrient concentrations first reached threshold concentrations. The nutricline pressure was converted to depth (m) using a UNESCO formula revised by Leroy & Parthiot (1998) (*9*).

Nutricline threshold concentrations

We defined nutricline depths where interpolated concentrations reached threshold values, a method that is commonly employed throughout oceanographic studies (*10 – 13*). Alternatively, nutricline depths can also be defined based on an inflection point in the nutrient depth profile (*14*). However, an accurate measure of the inflection point requires high vertical resolution of the nutrient profile since the profile shape can vary regionally and temporally (*15*). In contrast, referring to a threshold concentration requires fewer assumptions about the profile shape. Furthermore, efforts to quantify the mixed layer depth from density profiles show that referring to a threshold difference from the surface yields more consistent results than a gradient criterion (*16*). As surface nutrients are near zero in the oligotrophic ocean, threshold concentrations for nutricline depths also reflect threshold differences. Therefore, a threshold concentration can be used to indicate the nutricline depth, especially for profiles with lower vertical resolution.

Our analysis focuses on nutricline depths defined from 3 µmol kg^{-1} nitrate and the Redfield (1934) proportion equivalent for phosphate (i.e., 3/16 µmol kg^{-1}, assuming that N:P = 16:1) (*17*). We chose a threshold concentration for nitrate that was higher than other studies (*10 - 13*) to minimize influence from the surface mixed layer and ensure detectability of low phosphate concentrations (*18*, *19*). We chose a Redfield-equivalent value for phosphate since the Redfield ratio remains a well-established reference point for understanding nutrient dynamics (*20*), phytoplankton composition (*21*), and carbon cycling (*22*). However, we also quantified nutricline trends in the GO-SHIP data set using lesser ($[NO_3^-]$ = 1 µmol kg^{-1} and $[PO_4^{3-}]$ = 1/16 µmol kg^{-1}) and greater ($[NO_3^-]$ = 5 µmol kg^{-1} and $[PO_4^{3-}]$ = 5/16 µmol kg^{-1}) threshold concentrations. Pairing the lesser and greater threshold concentrations for nitrate and phosphate allowed us to also compare results across a broad range of N:P proportions from about 3 to 80. These additional analyses helped determine if the choice of threshold concentrations had a considerable effect on the nutricline trends.

Criteria for nutricline depths

We followed a series of criteria for quantifying nutricline depths and trends to improve the accuracy of our analyses. First, we only recorded the nutricline depth of a cast if there existed interpolated concentrations above and below said depth. Second, we rounded the geographic coordinates of each nutricline depth to the nearest degree of latitude and longitude to quantify average nutricline depths per unique site and year. Lastly, we quantified trends only from average nutricline depths that were deeper than 50 m and at a latitude from 45°S to 45°N. We also repeated our analysis using a depth boundary of 25 m. We selected these boundaries to target trends in regions where nutrients limit growth and minimize effects from seasonal changes in stratification (*23*). We address potential biases resulting from these spatial boundaries in the "Caveats and conclusions" section.

Significance tests for nutricline trends

We tested the significance of nutricline trends using two independent methods. First, we fitted linear regression models to average nutricline depths through time. These nutricline depths

were annual averages for each unique pair of geographic coordinates, or site. Two separate regressions were fitted to nitracline and phosphaclines, and an *F*-test determined if each regression was significant (i.e., $p < 0.05$). Second, we analyzed each unique site that had average nutricline depths recorded for two or more unique years. For each site, we fitted a regression to its average nutricline depths vs. years to quantify its site-specific trend. These site-specific trends were quantified separately for nitraclines and phosphaclines. The sign test determined if the global median site-specific trend was significantly different from zero. Additionally, the Kruskal-Wallis test determined if the median trends of nitracline and phosphaclines were significantly different from each other. We also calculated 95% confidence intervals ($CI_{95\%}$) for the median trends by generating 10,000 bootstrap samples of the data set. Thus, with global regressions and site-specific trends, we were able to test if nitracline and phosphacline depths were generally shifting in recent decades.

<u>Comparing trends from random populations</u>

In addition to the significance tests, we randomized sampling time of the nutriclines to further examine the robustness of the observed trends. We constructed 10,000 randomized conglomerate data sets by scrambling the years of nutricline depths, where each depth was an annual-average for a unique site. For each of these 10,000 random populations, we found the median values of the site-specific trends for nutricline depths. We recorded these median trends of random populations (MToRP) and determined the portion of MToRP with an absolute value greater than the absolute value of the observed median trend. Quantifying this portion of the MToRP distribution served as a two-tailed *p* value to test the significance of the observed median trend.

We also investigated how measurement error may have affected the nutricline trends. From an intercomparison study of nutrient detection, nitrate and phosphate concentrations had average standard deviations of about 400 nmol kg^{-1} and 50 nmol kg^{-1} respectively (*24*). We added additional error to the GO-SHIP observations by creating two normal distributions of values. Both distributions had means equal to zero, and standard deviations equal to twice the values from Aoyama et al. (2007) (i.e., 800 nmol kg^{-1} and 100 nmol kg^{-1}) (*24*). For every nutrient concentration in the observations, we randomly sampled a value from either the first or second distribution depending on whether the nutrient was nitrate or phosphate. We added this sampled value to the nutrient concentration to impose additional measurement error. We then quantified nutricline depths and trends after altering all concentrations. We then compared the median values and variability of the site-specific trends to those from the unaltered observations.

<u>Investigating biases with climatologies</u>

To explore potential temporal biases in the cruise observations, we analyzed nutrient concentrations from the National Oceanic and Atmospheric Administration's World Ocean Atlas data product version 2018 (WOA18). WOA18 is an objectively-analyzed global climatology of nutrient concentrations per 1° of latitude and longitude for every month (*25*). The vertical profiles reached down to 800 m deep with depth bin sizes ranging 5 - 50 m. For every unique pair of rounded geographic coordinates (i.e., "site"), we interpolated nitrate and phosphate concentrations per 1 m and found the average nutricline depths.

We investigated WOA18 to reveal if there was a notable bias in the months nutriclines were sampled. First, we indexed each nutricline depth based on the latitude, longitude, and month of the respective cast. We then replaced each observed nutricline depth with its respective monthly

value in WOA18. Next, we proceeded to find the annually-averaged nutricline depth for each unique site of each year. Then, we determined the trends of nutricline depths with time. If there were considerable seasonal biases in cruise casts, then the global medians of these trends would deviate from zero.

We also constructed random populations from WOA18 to determine how variability in site-specific trends affected their median values. First, we randomly selected a potential monthly nutricline depth for each unique site with nutricline depths observed for two or more years in the GO-SHIP collection. We then paired these to another random, potential monthly nutricline depth to simulate a 50-year trend for each site. We repeated this procedure until 10,000 random populations of the conglomerate data set were made. Second, we performed a similar procedure where we paired one set of monthly values to another set, but here we imposed a ubiquitous site-specific trend by changing the second set of monthly values by a rate equivalent to the median trend from observations. These two analyses determined if the median trend was controlled by monthly variability in sampling. Third, we imposed a variable site-specific trend by changing each monthly value in the second set by a rate of change randomly selected from a normal distribution of rates. We altered the mean and standard deviation of this normal distribution until the median trend and the variance of simulated nutricline depths (i.e., IQR) both matched the observations. This analysis determined if the median trend actually captured an underlying tendency of the site-specific trends despite their high variability. In summary, these analyses with WOA18 determined if variability in the site-specific trends had considerable influence on their median values.

Improved technology may have increased the accuracy of nutrient concentrations over time. To test this, we investigated GLODAPv2.2016, another mapped climatology of nutrient concentrations for each 1° of latitude and longitude (*2*, *26*). GLODAPv2.2016 provided nitrate and phosphate concentrations down to 5,500 m with depth bins ranging from 10 - 500 m. At each unique site, we linearly interpolated nutrient concentrations for every 1 m of depth. We then recorded the interpolated concentrations at the depths of 100, 300, 500, 1,000, 2,000, 3,000, and 4,000 m for each site. Next, we went through each cast of the GO-SHIP observations and recorded the interpolated concentrations at these same depths. For each cast, we found the difference of the observed concentrations and the mapped climatology at these specified depths. We then fitted linear regressions to the differences at each site. If improved technology imposed a measurement bias, then the differences at deeper depths may show a negative trend with time.

Assessing median trends with time-series validations

We investigated the long-term trends of nutricline depths from the sites for the Bermuda Atlantic Time-series Study (BATS) and A Long-term Oligotrophic Habitat Assessment (ALOHA), two open-ocean stations with multi-decadal data (*27*). These sites offer high temporal resolution considering that nutricline depths were sampled 2 to 33 times per year at BATS and 3 to 12 times per year at ALOHA from 1988 to 2022 (Fig. S8A-D). For both sites, we calculated every possible combination of 2 data points for nutricline depth vs. time and found the overall median of these potential trends (i.e., Sen's slope) (Fig. S8E-H). This median trend is consistent with a regression slope and offers a more precise estimation of the long-term trend with the bootstrapped confidence intervals (Fig. S8I, J). Neither site shows faster phosphacline deepening, but this is within expectations since both are in the northern hemisphere and the global trends represent central tendencies rather than ubiquitous trends. Additionally, the site trends from two 5° x 5° grids of the global data (WOD) centered at BATS and ALOHA shows median values that

are consistent with each time-series (Fig. S8I, J). Therefore, the time-series data suggest that the median values of individual trends can reflect long-term changes in nutricline depths.

The time-series data also provide evidence that both hemispheres and all five subtropical gyres have enough site trends to reliably estimate their long-term changes. We randomly sampled 100 potential trends of each time-series and found that the true median trend was consistently captured within each subset median's confidence interval (Fig. S8I, J). Given that each subset had a smaller sample size with larger variance ($n = 100$, $IQR \geq 5.0$) than any regional trends ($n \geq 130$, $IQR < 5.0$), this suggests that the confidence interval of each region also captures its long-term trend. Additionally, this also supports that the long-term trend of each region can be captured from sites with as few as 2 years of nutricline depths sampled (i.e., 2 data points) throughout the 51-year period. Therefore, despite varying degrees of data sparsity, the time-series data suggests that there are sufficient data to perform regional comparisons for nutricline trends.

Testing trend robustness by filtering out observations

Sampling density with depth had limited impact on the median trends (Fig. S9A-C). We filtered the global observations based on the number of unique depths nutrients were sampled per cruise cast. The nitracline median trend becomes insignificant from zero after removing casts with less than 6 depths sampled (4% of casts removed). The nitracline and phosphacline median trends became insignificantly different from each other after removing casts with less than 30 depths sampled (70% of casts removed). The phosphacline median consistently shows deepening until casts with less than 37 depths sampled are removed (97% of casts removed). This extensive cast filtering demonstrates the robustness of phosphacline deepening.

We also filtered the global observations based on the number of unique years sampled per site, and came to similar conclusions about the data (Fig. S9D-F). The nitracline median trend became insignificantly different from zero after removing sites with less than 3 years of nutricline depths (51% of sites removed). The difference between the median trends became insignificantly different from each other after removing sites with less than 6 years of nutricline depths (93% of sites removed). The phosphacline median continued to indicate deepening until sites with less than 7 years were removed (96% of sites removed). Again, the substantial amount of site filtering required to render the trends insignificant highlights the robustness of faster phosphacline deepening.

We also filtered out site trends based on what the time-series data suggest are feasible, and found again that our findings are robust. The time-series data indicate that a long-term trend of nutricline depths over multiple decades can be as large as 2 m per year (Fig. S8). Considering this, we filtered out any nutricline trend in the global data that was less than -2 m/yr or greater than 2 m/yr. This results in the global nitracline median becoming insignificantly different from zero (-0.3 m/yr, $CI_{95\%}$ = [-0.10, 0.05]), but the phosphacline median remains indicative of deepening (0.23 m/yr, $CI_{95\%}$ = [0.09, 0.34]). Therefore, the global tendency of phosphacline deepening is not dependent on seemingly unrealistic trends.

CMIP6 predicted nutriclines

The Coupled Model Intercomparison Project Phase 6 (CMIP6) is a collection of global models that each predict biogeochemical cycles with time (*28*). Séférian et al. (2020) outline some key model differences that contribute to their contrasting predictions of biogeochemistry. These models differ in their ocean-climate interactions, organic matter cycling rates, marine plankton communities, biological processes, nutrient ratios, and more (*28*).

Here, we analyzed nutrient concentrations from CMIP6 under three emission scenarios: the historical period, middle-of-the-road (SSP2-4.5), and business-as-usual (SSP5-8.5) (Table S6) (*29*, *30*). For the historical scenario, we confined the years to 1972 - 2014 to better match the time span of the observations. The future emission scenarios were confined to the years 2015 - 2100. Our primary variables of interest were marine concentrations of nitrate and phosphate ("no3" and "po4" respectively). We also analyzed sea temperature ("thetao") and salinity ("so") through time to convert the nutrient concentrations from mol m^{-3} to µmol kg^{-1}, and determine trends in nutricline densities. These variables came from consistent model variants in terms of realization, initialization, method, physics, and forcing; the "r1i1p1f1" variant was used when available. With these variables, we quantified nitracline and phosphacline depths with time.

Nutricline depths in CMIP6 were quantified similar to the cruise observations. For each model grid point, we linearly interpolated nutrient concentrations for every 1 dbar of pressure up to 1000 dbar. Pressure was converted to meters of depth using a UNESCO formula revised by Leroy & Parthiot (1998) (*9*). Model grid points were then rounded to the nearest degree of latitude and longitude to quantify average nutricline depths and densities for each unique site and year. We quantified trends for each site from 45°S - 45°N that had average nutricline depths deeper than 50 m for two or more unique years. Lastly, we tested if trends in vertically-integrated ocean nitrogen fixation rates ("intpn2") were related to predicted nutricline trends. Accordingly, we determined if CMIP6 was consistent with our hypothesis that nitrogen fixation rates together with stratification drive the decline of phosphate-to-nitrate availability.

CESM2 nutrient dynamics and iron deposition (D_{Fe}) experiments

We explored nutrient dynamics and the effects of iron deposition in an updated version of Community Earth System Model version 2 – Marine Biogeochemistry Library (CESM2-MARBL). CESM2-MARBL encompasses many essential features of marine ecosystems, including major limiting macronutrients (N, P, Si), iron cycling processes, variable nutrient assimilation ratios, mineral ballasting, a Q10 growth parameterization, and dynamic dissolved organic matter (DOM) cycling (*31*). While the standard version of MARBL has three phytoplankton types (small phytoplankton, diazotrophs and diatoms) and one zooplankton, we used an updated version of MARBL in this study which includes eight types of phytoplankton and four types of zooplankton (MARBL-8P4Z) (*32*). For phytoplankton, the 8P4Z model includes 1) pico-sized phytoplankton (0.5-2.0 µm): *Prochlorococcus*, *Synechococcus*, pico-eukaryotes and nitrogen-fixing diazotrophs; 2) nano-sized phytoplankton: *Phaeocystis* (2-10 µm), coccolithophores (5-10 µm) and generic other nanophytoplankton (2-200 µm); 3) micro-sized phytoplankton (20-200 µm): diatoms. For zooplankton, the 8P4Z model includes small microzooplankton (5-20 µm, ciliates, nanoflagellates), large microzooplankton (20-200 µm, copepod nauplii, small dinoflagellates etc.), mesozooplankton (200-2000 µm, smaller copepod, large dinoflagellates) and macrozooplankton (>2000 µm, larger copepod, krill). Besides the modified plankton types, the 8P4Z model also has a better representation of the phytoplankton group-specific variable stoichiometries, both for iron (*33*) and for the macronutrients (N, P, Si) (*34*).

To begin our analyses in CESM2-MARBL-8P4Z, we initialized the simulation with JRA55-do (*35*) interannual temperature forcing (1958-2018, 61 years) for five repeated cycles (305 years in total) (*31*). We then ran a control run after the 305-year initialization, where historical emissions continued for another 61 years. From this control run, we investigated the relationships between nutricline depths and nutrient vertical flux. Given the extreme difficulty in observing nutrient

inputs into the upper ocean, we instead referred to the organic matter export at 100 m depth, which is indicative of the supply flux of upper ocean nutrients under steady-state conditions (*36*). We also examined the effect of using different threshold concentrations by finding the Spearman's rank correlation coefficient and root mean squared error (RMSE) of each relationship (Fig. S2). To quantify nutricline depths, nutrient concentrations were converted to μmol kg^{-1} and nutricline depths were quantified using the same method as the CMIP6 models.

We then ran experimental runs where we tested how nitrogen fixation rates and nutricline depths responded to atmospheric iron deposition (D_{Fe}) increasing globally, in just the northern hemisphere, or in just the southern hemisphere. We augmented D_{Fe} in these areas by either 25%, 50%, or 100%. Each experimental run started immediately after the 305-year initialization and lasted for 61 years as well. Therefore, a total of 9 experimental runs were conducted to constrain how D_{Fe} affects nitrogen fixation rates and nutricline trends in CESM2.

We referred to other renditions of CESM2 to determine if flexible C:P in phytoplankton affected nutricline trends, which essentially tested if flexible phosphate uptake rates had any effect. We analyzed two experimental scenarios from Kwon et al. (2022), one where phytoplankton C:P is fixed at Redfield proportions (16:1), and another where phytoplankton C:P increased with declining phosphate concentrations as a power-law function (*37*). These experiments were conducted under historical and SSP3-7.0 emission scenarios. For these experiments, we quantified nutricline depths using the same method as the CMIP6 models, with the exception that nutrient concentrations were left in units of mmol m^{-3}. Therefore, the nitracline and phosphacline were defined based on 3 mmol m^{-3} and 3/16 mmol m^{-3}, respectively.

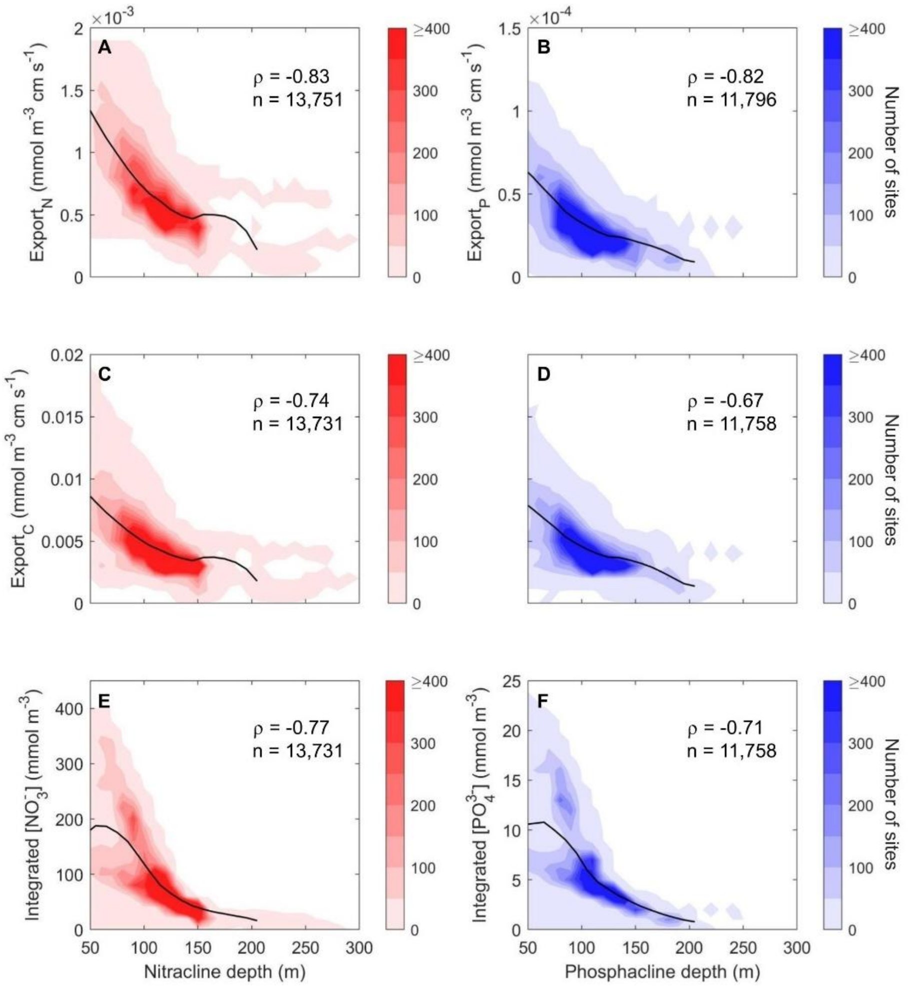

Figure S1: Nutricline depths showed strong negative correlations with nutrient supply and broader ecosystem dynamics in an Earth System Model. Each data point was the mean values of each site (i.e., one pair of rounded geographic coordinates) in the CESM2-MARBL-8P4Z model. We only used mean nutricline depths deeper than 50 m and between 45°S – 45°N to assess these relationships. (**A**, **B**) Nitracline and phosphacline depths were linked with organic nitrogen and phosphorus export out of the upper ocean ($Export_N$ & $Export_P$) respectively. As the surface concentration is close to zero, there is little advection of pre-formed nutrients. Hence, the output fluxes approximate nutrient input fluxes into the upper ocean under steady-state conditions (*36*). (**C**, **D**) The nutricline depths also reflected organic carbon export ($Export_C$), indicating their link to broader ecosystem dynamics like carbon sequestration. (**E**, **F**) We confirmed that nutricline depths also reflect nutrient inventory by integrating all nutrient concentrations above 200 m. The correlation strengths were calculated as the Spearman's rank correlation coefficients (ρ) and the black lines are moving medians with bins spaced every 10 m.

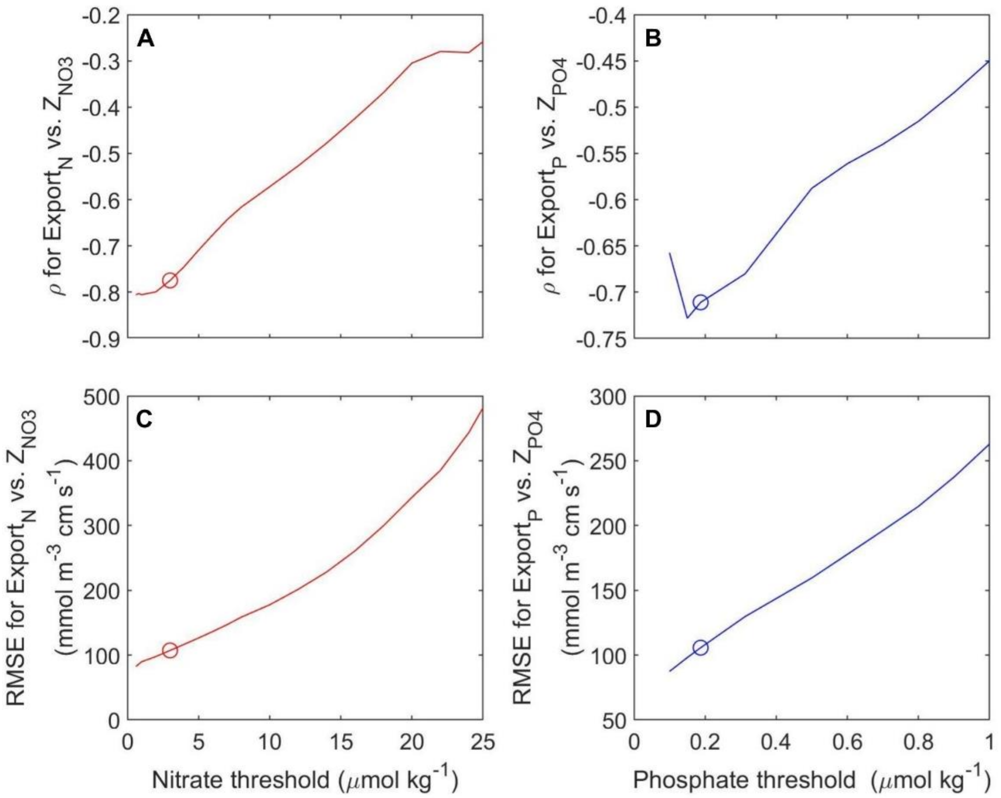

Figure S2: The featured threshold concentrations yielded nutricline depths that were some of the best proxies for nutrient dynamics compared to other thresholds. We quantified nutricline depths in the CESM2-MARBL-8P4Z model using a range of different concentration thresholds and related these depths to organic matter export at 100 m depth, the nutrient output flux for the upper ocean. This flux is indicative of the nutrient supply flux under steady-state conditions (*36*). (**A**, **B**) The Spearman's rank correlation coefficient for export vs. nutricline depths, and (**C**,**D**) the root mean squared errors for these relationships. We only used mean nutricline depths deeper than 50 m and between 45°S – 45°N to assess these relationships. We selected the range from lowest to highest thresholds based on reliability from measurement error (*24*) and the mean nutrient concentrations presumably below the nutricline at 500 m depth. The circles indicate the values from our featured threshold concentrations ($[NO_3^-]$ = 3 µmol kg^{-1} & $[PO_4^{3-}]$ = 3/16 µmol kg^{-1}).

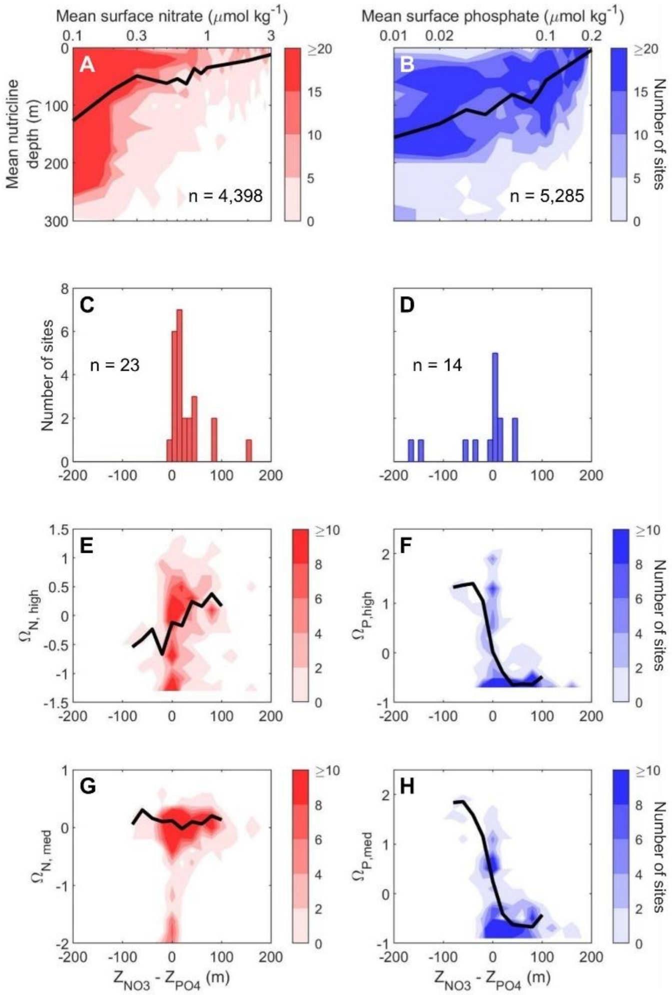

Figure S3: Observed nutricline depths were linked to observed nutrient availability and limitation. The first row shows mean values (1972 - 2022) of (**A**) nitracline and (**B**) phosphacline depths vs. mean values of surface nutrient concentrations for each unique pair of geographic coordinates, or site (n). Nutricline depths were determined from GO-SHIP using threshold concentrations of [NO_3^-] = 3 µmol kg^{-1} and [PO_4^{3-}] = 3/16 µmol kg^{-1}. The black lines are moving medians with bins that were logarithmically-spaced from 10^{-2} to 10^{1} µmol kg^{-1}. The second row shows mean values of Z_{NO3} - Z_{PO4} for each site where there was (**C**) nitrogen limitation without phosphorus limitation, and (**D**) phosphorus limitation determined by bioassay experiments conglomerated by Browning & Moore (2023) (*38*). The third and fourth rows are mean values of Z_{NO3} - Z_{PO4} for each site (n = 353) where there was (**E**) high nitrogen stress, (**F**) high phosphorus stress, (**G**) medium nitrogen stress, and (**H**) medium phosphorus stress determined by *Prochlorococcus* genetic biomarkers in Ustick et al. (2021) (*39*). The black lines are moving medians with bins spaced every 10 m.

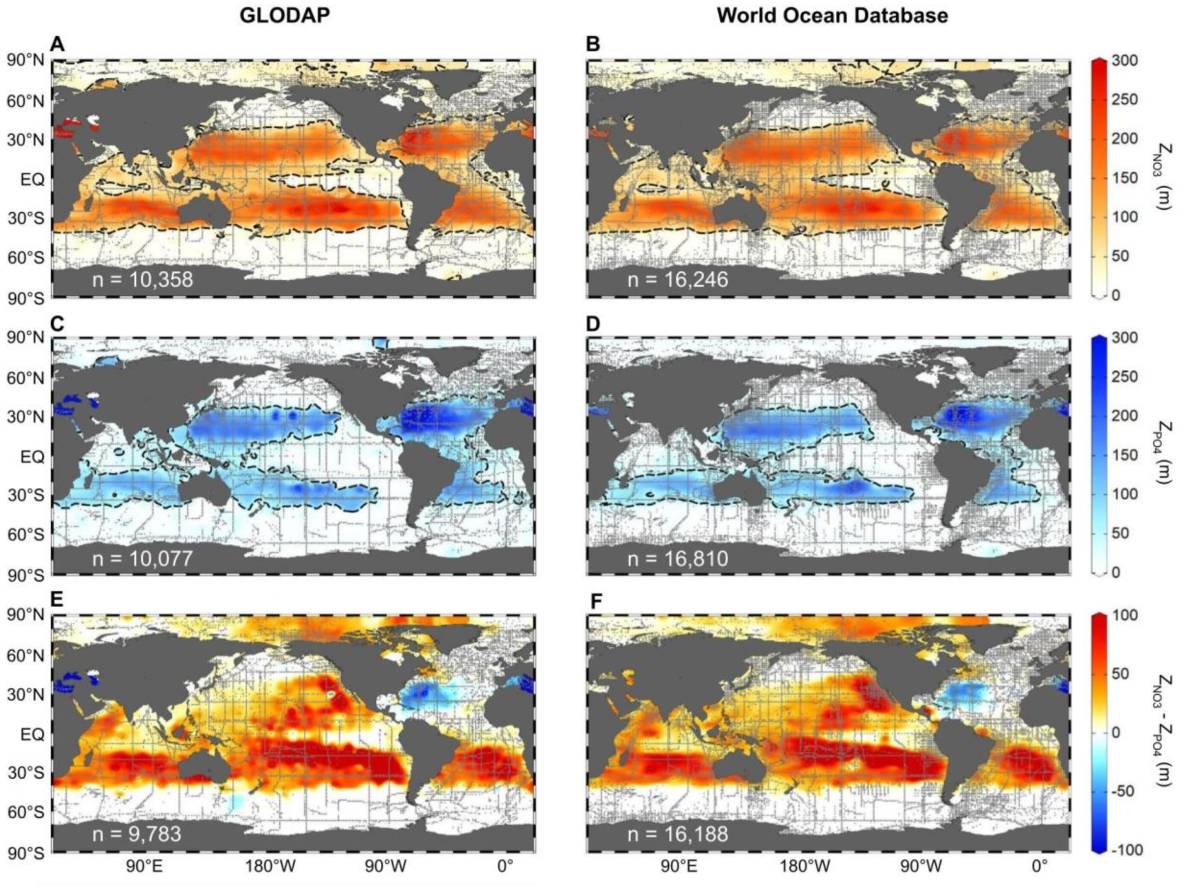

Figure S4: Mean nutricline depths from other conglomerate data sets were consistent with GO-SHIP. (**A**,**B**) Average nitracline depths (Z_{NO3}), (**C**,**D**) average phosphacline depths (Z_{PO4}), and (**E**,**F**) the difference of average nitracline and phosphacline depths (Z_{NO3} - Z_{PO4}) from the GLODAPv2.2022 merged and adjusted data product and the World Ocean Database (WOD), respectively. Z_{NO3} and Z_{PO4} were defined with threshold concentrations of $[NO_3^-]$ = 3 µmol kg^{-1} and $[PO_4^{3-}]$ = 3/16 µmol kg^{-1}, respectively. Here, Z_{NO3} and Z_{PO4} were averaged from 1972 to 2022 for each unique pair of geographic coordinates (gray dots with sample size "n"). The dashed line is the contour at 50 m depth. The depth differences were calculated using the averaged depths.

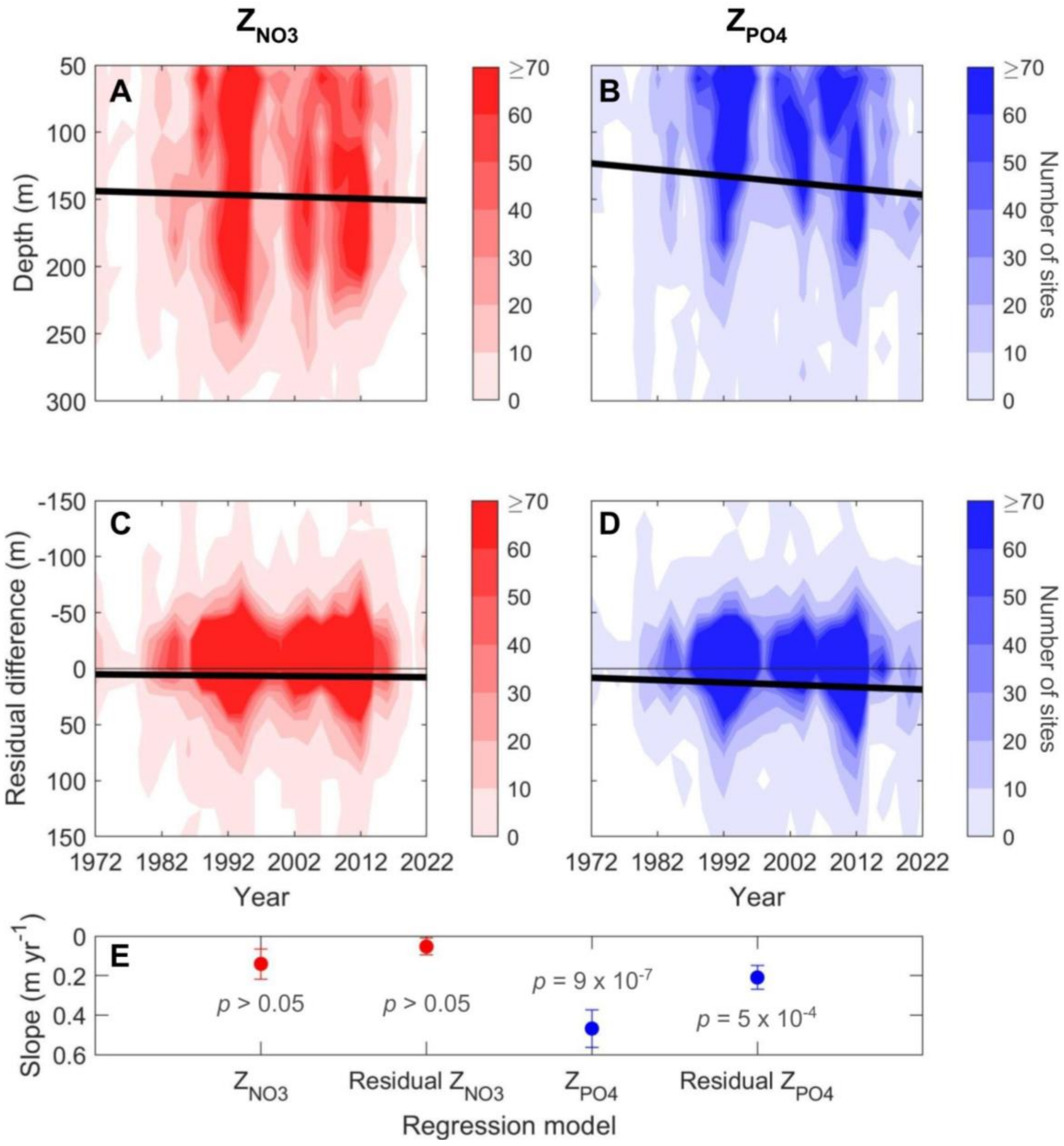

Figure S5: Global regressions show a faster deepening of Z_{PO4} relative to Z_{NO3}. Here, Z_{NO3} and Z_{PO4} were average values from the GO-SHIP data set for each unique site and year. They were defined with threshold concentrations of $[NO_3^-] = 3$ µmol kg^{-1} and $[PO_4^{3-}] = 3/16$ µmol kg^{-1}. Linear regressions fitted to (**A**) Z_{NO3} with time (n = 7,513) and (**B**) Z_{PO4} with time (n = 6,823). We also subtracted the WOA18 monthly nutricline depths from observations to fit linear regressions to the residual differences of (**C**) Z_{NO3} (n = 7,680) and (**D**) Z_{PO4} (n = 6,365) with time. (**E**) The slopes of each regression model with their respective p values. The error bars are their standard errors.

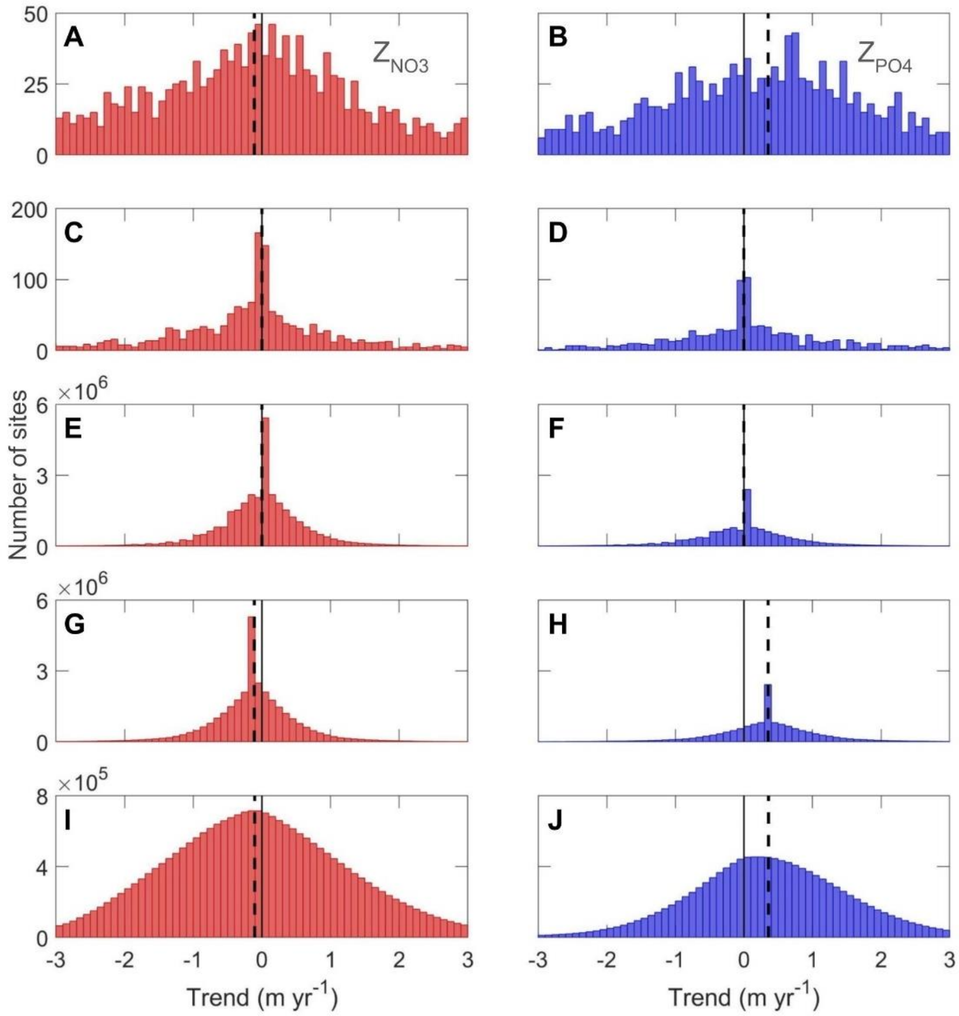

Figure S6: World Ocean Atlas shows that the median trends capture the underlying tendency of site-specific trends. (**A**,**B**) Observed site-specific trends of nitracline and phosphacline depths from GO-SHIP. (**C**,**D**) Site-specific trends after substituting GO-SHIP observations with their respective monthly values in World Ocean Atlas version 2018 (WOA18). (**E**,**F**) Simulated site-specific trends of 10,000 random populations of paired WOA18 monthly values. (**G**,**H**) Simulated site-specific trends of 10,000 random populations after imposing a ubiquitous trend on WOA18 monthly values. The ubiquitous trend was equivalent to the observed median trend from GO-SHIP. (**I**,**J**) Simulated site-specific trends of 10,000 random populations after imposing a variable site-specific trend on WOA18 monthly values. Each site-specific trend came from a normal distribution with a mean and standard deviation selected such that the median trend and variance in depths were the same as the GO-SHIP observations. The dashed lines mark the median values.

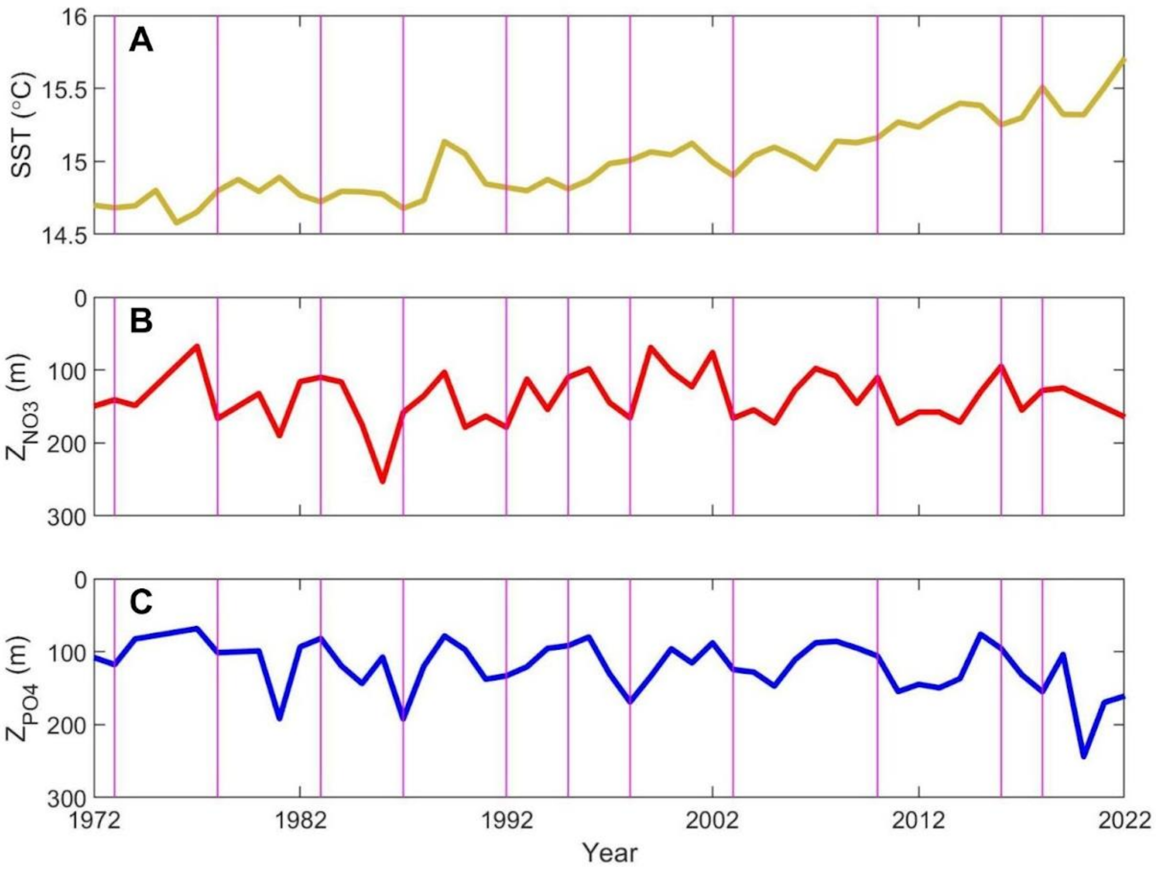

Figure S7: The ENSO cycle and other climate phenomena may affect interannual nutricline variance. Annual median values of (**A**) sea surface temperature (*40*), (**B**) nitracline depth, and (**C**) phosphacline depth. Nutricline depths were quantified from the GO-SHIP data set using threshold concentrations of [NO_3^-] = 3 µmol kg^{-1} and [PO_4^{3-}] = 3/16 µmol kg^{-1}. The magenta lines denote El Niño years determined by the Ensemble Oceanic Niño Index (*41*).

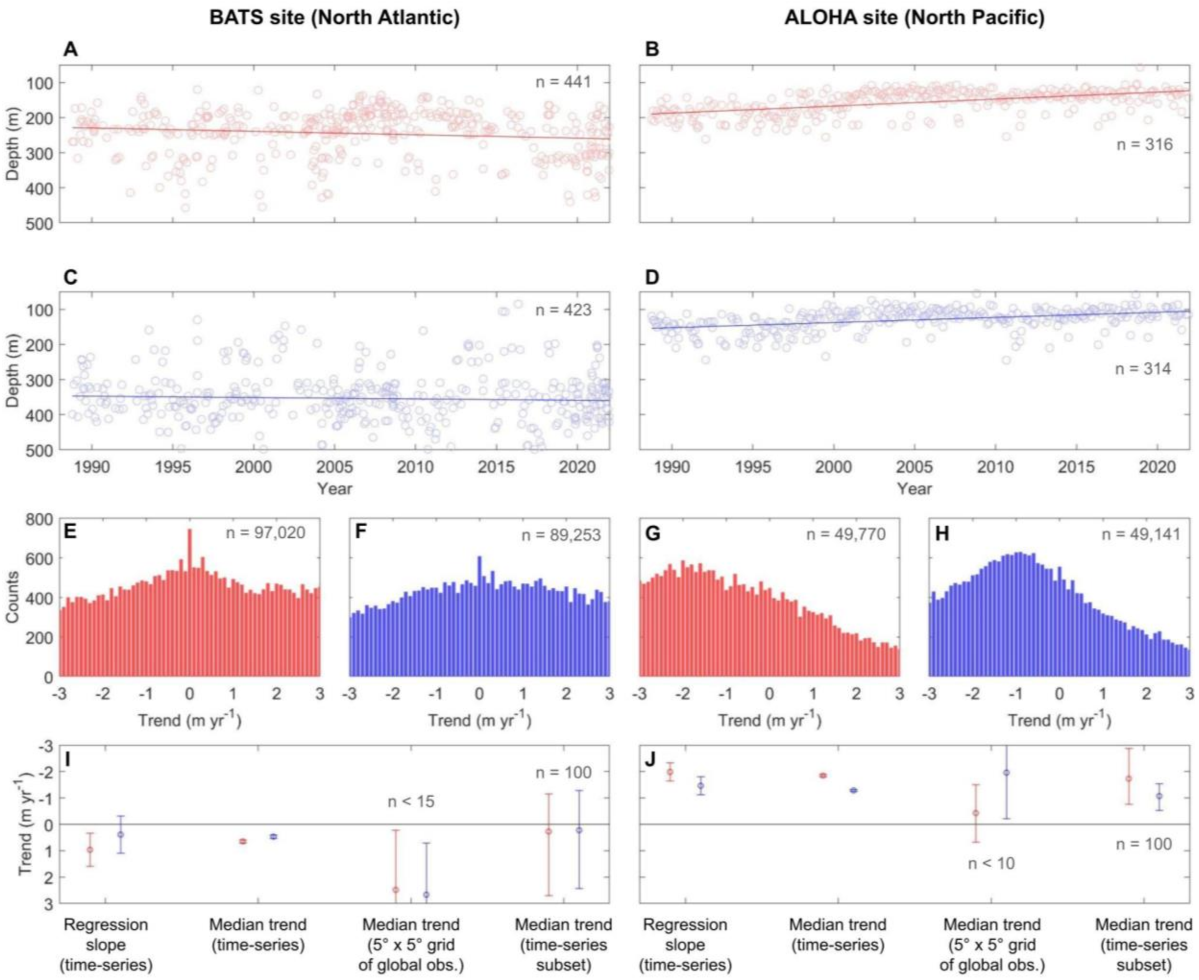

Figure S8: Two time-series sites suggest that both hemispheres and all five subtropical gyres have sufficient data to reliably estimate their long-term changes. (**A**,**B**) The first row shows nitracline depths (red) over time from the sites for the Bermuda Atlantic Time-series Study (BATS) and A Long-term Oligotrophic Habitat Assessment (ALOHA) (*27*). (**C**,**D**) The second row is phosphacline depths (blue) over time from these sites. The third row depicts histograms of potential trends for every possible combination of two data points from (**E**, **F**) BATS and (**G**, **H**) ALOHA. The fourth row shows long-term trends for (**I**) BATS and (**J**) ALOHA calculated from regression slopes and median trends. The median trends from the time-series are based on potential trends from a single site, while the median trends from the global observations are calculated from trends across multiple sites. The global observations are confined to grids centered at each time-series site, and the data is from the World Ocean Database (WOD). The error bars are the 95% confidence intervals, which were calculated for each median by generating 10,000 bootstrap samples.

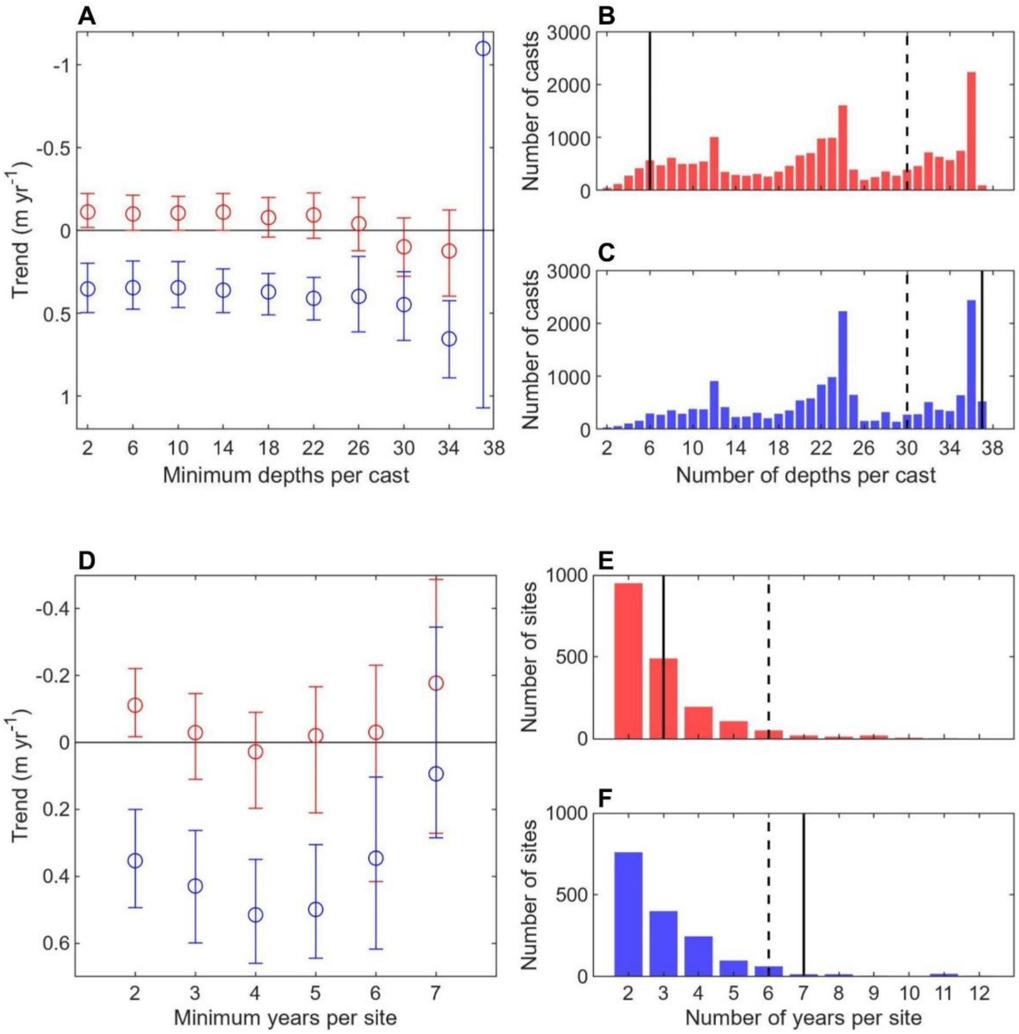

Figure S9: The global nutricline trends remain robust even after extensive filtering based on sampling density. (**A**) Nitracline (red) and phosphacline (blue) trends after removing cruise casts sequentially based on the number of depths that nutrient concentrations were sampled. The circles indicate median trends and the error bars are their 95% confidence intervals from 10,000 bootstrapped samples. (**B**, **C**) Histograms showing the number of casts based on the number of depths sampled. The solid line marks where the median trend became insignificantly different from zero, and the dashed line denotes where the nitracline and phosphacline median trends became insignificantly different from each other. (**D**) Nitracline and phosphacline trends after removing sites sequentially based on the number of years sampled. (**E**, **F**) Histograms showing the number of sites based on the number of years available. The circles, error bars, solid lines, and dashed lines follow the same interpretation as in (A-C). This figure shows nutricline data from GO-SHIP using threshold concentrations of $[NO_3^-]$ = 3 μmol kg^{-1} and $[PO_4^{3-}]$ = 3/16 μmol kg^{-1}.

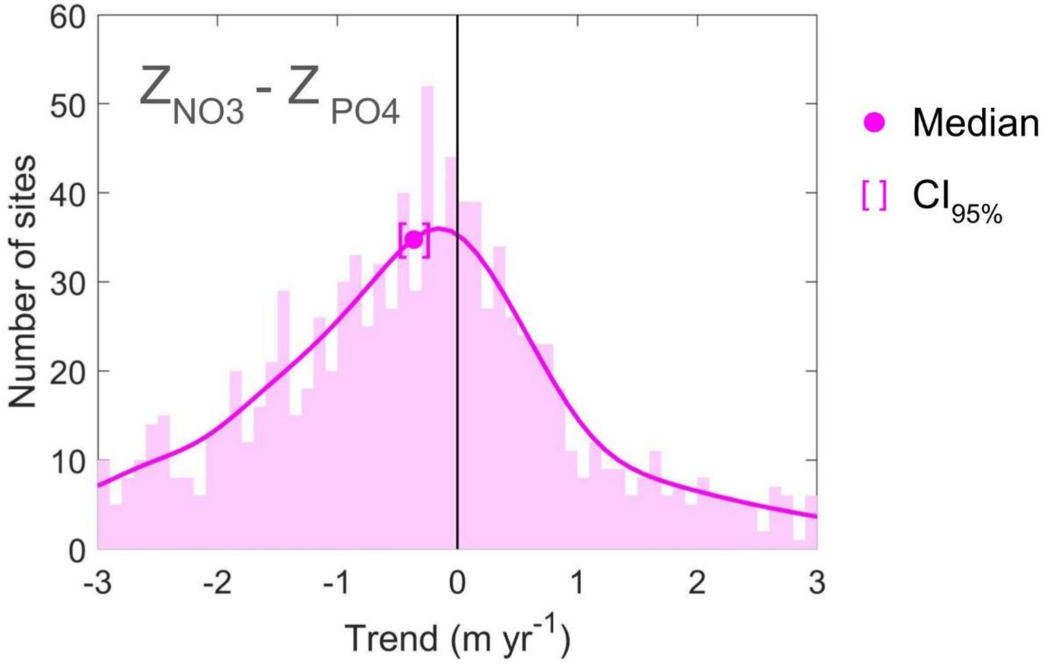

Figure S10: The median trend of Z_{NO3} - Z_{PO4} was negative, suggesting Z_{PO4} deepened faster. Z_{NO3} and Z_{PO4} were defined from the GO-SHIP data set with threshold concentrations of $[NO_3^-]$ = 3 µmol kg^{-1} and $[PO_4^{3-}]$ = 3/16 µmol kg^{-1} respectively. The difference was found using annual averages of Z_{NO3} and Z_{PO4}, such that they were deeper than 50 m deep and from 45°S - 45°N. The difference was calculated for each unique site and year from 1972 - 2022.

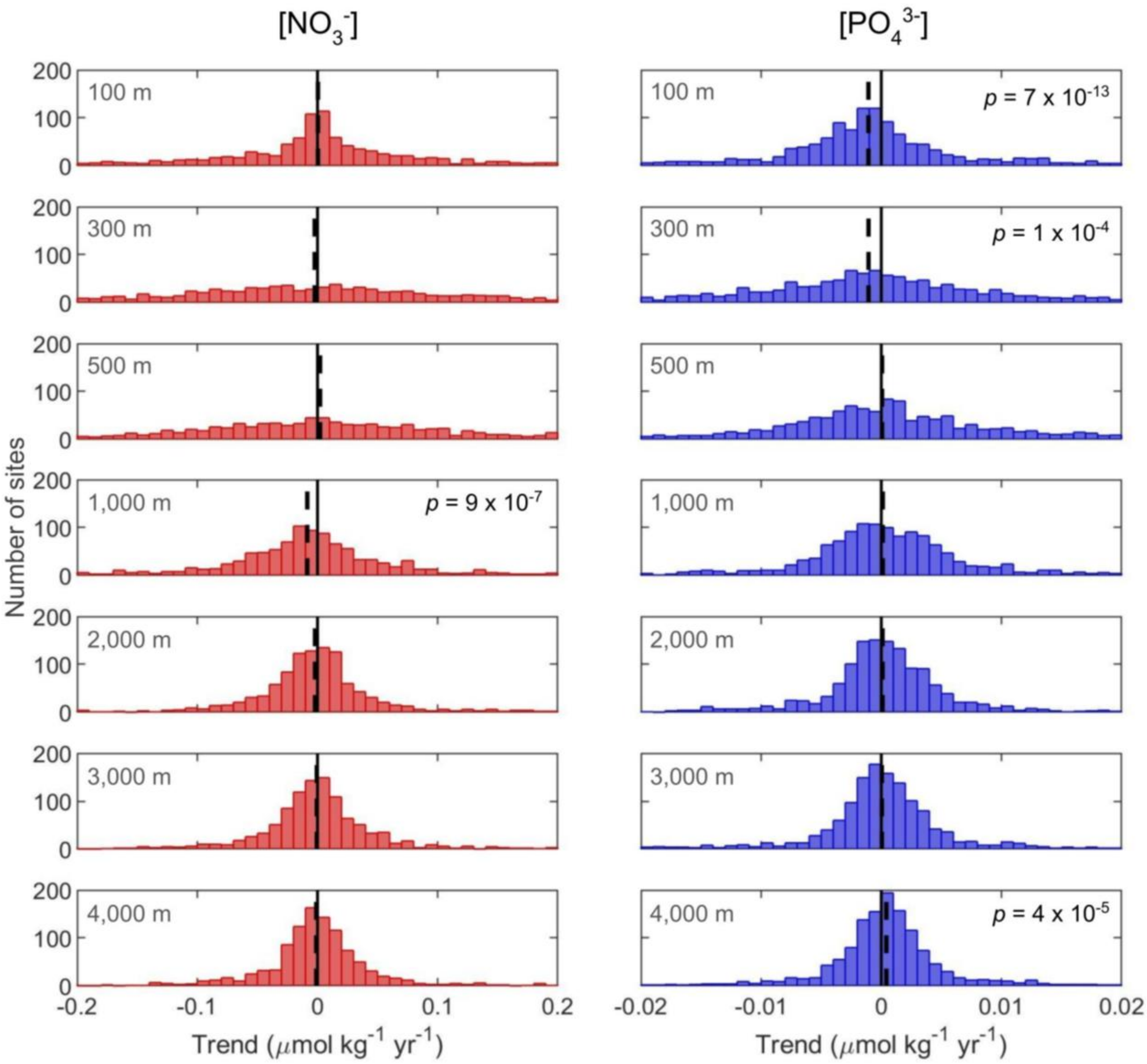

Figure S11: The difference of GO-SHIP observations and GLODAP climatology showed limited measurement bias. These histograms show the trends with time for the differences between observed nutrient concentrations in GO-SHIP and their respective climatology values in GLODAPv2.2016. Observed concentrations were averaged to each unique site and year. The p values from the sign test that were greater than 0.05 are not displayed.

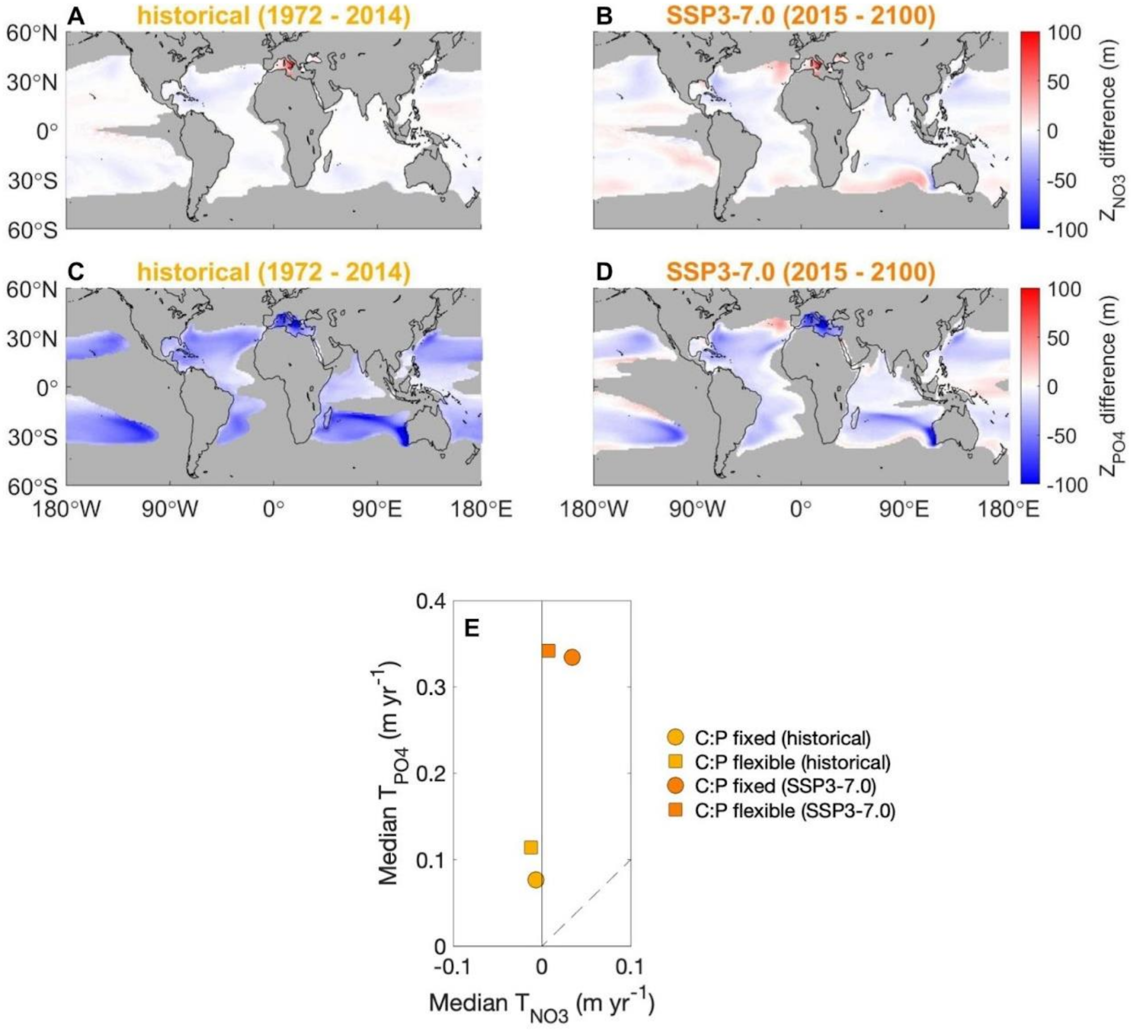

Figure S12: Flexible phytoplankton C:P in CESM2 shoaled mean phosphacline depths. We determined how flexible phosphate uptake affected nutricline trends in a CESM2 model described by Kwon et al. (2022) (*37*). The difference of mean Z_{NO3} with flexible C:P and mean Z_{NO3} with fixed (i.e., Redfield) C:P under the (**A**) historical and (**B**) SSP3-7.0 scenarios. The difference of mean Z_{PO4} with flexible C:P and mean Z_{PO4} with fixed C:P under the (**C**) historical and (**D**) SSP3-7.0 scenarios. Z_{NO3} and Z_{PO4} were defined with threshold concentrations of $[NO_3^-]$ = 3 mmol m^{-3} and $[PO_4^{3-}]$ = 3/16 mmol m^{-3}, respectively. The difference calculation uses mean values of nutricline depths over the entire time period. Negative values indicate that average nutricline depths were deeper when C:P was fixed, whereas positive values indicate they were deeper when C:P was flexible. (**E**) Median site-specific trends of nitracline and phosphaclines for fixed and flexible C:P under both emission scenarios. The dashed line is where $T_{PO4} = T_{NO3}$.

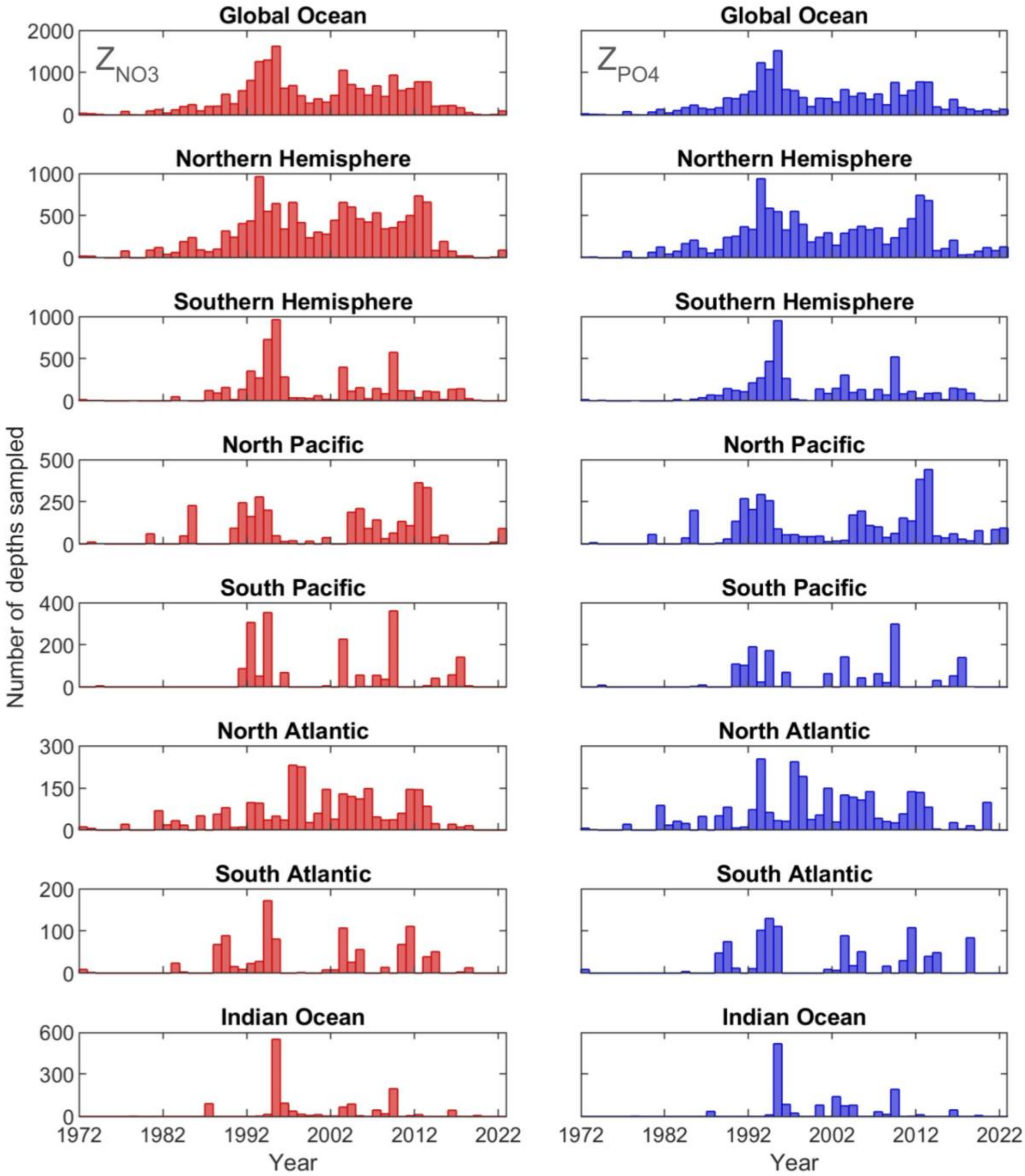

Figure S13: Z_{NO3} and Z_{PO4} were sampled concurrently throughout the global ocean. The histograms show the number of nitracline and phosphacline depths defined for each year. Each nutricline depth was an annual average for a unique site. The nutricline depths shown here were determined from the GO-SHIP data set using threshold concentrations of $[NO_3^-] = 3$ µmol kg^{-1} and $[PO_4^{3-}] = 3/16$ µmol kg^{-1} respectively. The boundaries defining each ocean region are described in Tables S2 and S4.

Table S1: Global regressions of nutricline depths

Data set	$[NO_3^-]$ threshold (μmol kg^{-1})	$[PO_4^{3-}]$ threshold (nmol kg^{-1})	Z_{NO3} Slope (m yr^{-1})	Z_{NO3} Slope SE (m yr^{-1})	Z_{NO3} Model *p* value	Z_{PO4} Slope (m yr^{-1})	Z_{PO4} Slope SE (m yr^{-1})	Z_{PO4} Model *p* value	ANCOVA *p* value
GO-SHIP	1	63	0.06	0.05	0.2 (n = 6,077)	0.13	0.09	0.2 (n = 2,977)	0.5
GO-SHIP	3	188	0.14	0.08	0.07 (n = 7,513)	0.47	0.10	9×10^{-7} (n = 6,823)	7×10^{-3}
GO-SHIP	5	313	0.35	0.10	7×10^{-4} (n = 8,166)	0.80	0.10	6×10^{-17} (n = 8,345)	2×10^{-3}
GO-SHIP (Z > 25 m)	1	63	0.25	0.06	3×10^{-5} (n = 7,071)	0.33	0.09	2×10^{-4} (n = 3,776)	0.4
GO-SHIP (Z > 25 m)	3	188	0.21	0.08	9×10^{-3} (n = 8,237)	0.53	0.09	6×10^{-9} (n = 7,736)	6×10^{-3}
GO-SHIP (Z > 25 m)	5	313	0.47	0.11	9×10^{-6} (n = 8,724)	0.82	0.10	5×10^{-18} (n = 9,085)	0.01
GO-SHIP (1982 - 2012)	3	188	0.30	0.10	3×10^{-3} (n = 6,510)	0.63	0.14	3×10^{-6} (n = 5,637)	0.04
GLODAP	3	188	0.27	0.06	2×10^{-6} (n = 11,364)	0.58	0.07	6×10^{-15} (n = 8,559)	5×10^{-4}
WOD	3	188	0.18	0.05	1×10^{-4} (n = 19,335)	0.35	0.06	1×10^{-9} (n = 13,441)	0.02

Each datapoint of a regression was an annually-averaged nutricline depth for a unique pair of geographic coordinates vs. the year. These depths were from 45°S – 45°N, deeper than 50 m depth, and from years 1972 – 2022 unless otherwise stated in the "Data set" column. The two groups for the ANCOVA had the same sample sizes as the regression models.

Table S2: Regional depths from GO-SHIP

Region	Spatial bounds	Mean Z_{NO3} (m)	Mean Z_{PO4} (m)	Mean Z_{NO3} - Z_{PO4} (m)
Global	-45°N to 45°N	123 (n = 9,317)	91 (n = 9,847)	31 (n = 9,189)
North hemisphere	1°N to 45°N	127 (n = 5,258)	116 (n = 5,416)	11 (n = 5,200)
South hemisphere	-45°N to -1°N	121 (n = 3,886)	62 (n = 4,257)	57 (n = 3,817)
North Pacific	15°N to 45°N, 120°E to -100°E	146 (n = 2,016)	116 (n = 2,091)	29 (n = 2,006)
North Atlantic	15°N to 45°N, -75°E to -10°E	138 (n = 1,509)	148 (n = 1,556)	-11 (n = 1,498)
South Pacific	-45°N to -15°N, 120°E to -70°E	155 (n = 1,181)	62 (n = 1,461)	86 (n = 1,181)
South Atlantic	-45°N to -15°N, -70°E to 20°E	119 (n = 844)	67 (n = 930)	53 (n = 842)
Indian Ocean	-45°N to -15°N, 30°E to 115°E	128 (n = 870)	75 (n = 855)	57 (n = 826)

Mean values of annually-averaged nutricline depths in the GO-SHIP data set from 1972 – 2022. The nitracline (Z_{NO3}) and phosphacline (Z_{PO4}) were defined from threshold concentrations of 3 µmol kg^{-1} and 3/16 µmol kg^{-1} for nitrate and phosphate respectively.

Table S3: Site-specific trends of nutricline depths

Data set	$[NO_3^-]$ threshold (μmol kg^{-1})	$[PO_4^{3-}]$ threshold (nmol kg^{-1})	T_{NO3}				T_{PO4}				Kruskal-Wallis test *p* value
			Median (m yr^{-1})	$CI_{95\%}$ (m yr^{-1})	MToRP two-tailed *p* value	Sign test *p* value	Median (m yr^{-1})	$CI_{95\%}$ (m yr^{-1})	MToRP two-tailed *p* value	Sign test *p* value	
GO-SHIP	1	63	-0.17	-0.27, -0.04	1×10^{-3} (n = 4,374)	5×10^{-3} (n = 1,544)	0.37	0.22, 0.50	0 (n = 2,197)	3×10^{-4} (n = 683)	9×10^{-7}
GO-SHIP	3	188	-0.11	-0.22, -0.02	0.02 (n = 5,539)	0.02 (n = 1,859)	0.35	0.20, 0.49	0 (n = 5,279)	3×10^{-6} (n = 1,641)	8×10^{-7}
GO-SHIP	5	313	-0.10	-0.21, 0	0.09 (n = 5,980)	0.05 (n = 1,974)	0.25	0.11, 0.37	0 (n = 6,463)	9×10^{-5} (n = 1,975)	2×10^{-5}
GO-SHIP (Z > 25 m)	1	63	-0.18	-0.28, -0.05	5×10^{-4} (n = 5,073)	2×10^{-3} (n = 1,771)	0.45	0.32, 0.62	0 (n = 2,801)	5×10^{-6} (n = 878)	4×10^{-9}
GO-SHIP (Z > 25 m)	3	188	-0.16	-0.27, -0.05	8×10^{-4} (n = 6,026)	2×10^{-3} (n = 2,020)	0.28	0.14, 0.43	0 (n = 5,949)	2×10^{-4} (n = 1,838)	1×10^{-7}
GO-SHIP (Z > 25 m)	5	313	-0.14	-0.23, -0.04	0.01 (n = 6,333)	8×10^{-3} (n = 2,099)	0.22	0.10, 0.33	0 (n = 6,960)	2×10^{-4} (n = 2,139)	1×10^{-5}
GO-SHIP (1982 - 2012)	3	188	-0.36	-0.50, -0.20	0 (n = 4,521)	3×10^{-6} (n = 1,662)	0.14	0.03, 0.34	0.04 (n = 4,113)	0.01 (n = 1,450)	2×10^{-5}
GLODAP	3	188	-0.13	-0.21, -0.05	4×10^{-4} (n = 9,174)	2×10^{-3} (n = 2,410)	0.27	0.15, 0.40	0 (n = 6,892)	2×10^{-7} (n = 1,800)	7×10^{-9}
WOD	3	188	-0.13	-0.20, -0.05	0 (n = 15,678)	2×10^{-4} (n = 3,781)	0.24	0.14, 0.35	0 (n = 10,882)	2×10^{-6} (n = 2,627)	6×10^{-10}

Site-specific trends were determined from annually-averaged nutricline depths that were from 45°S – 45°N, deeper than 50 m depth, and from years 1972 – 2022 unless otherwise stated in the "Data set" column. The 95% confidence intervals ($CI_{95\%}$) of the medians were calculated by generating 10,000 bootstrap samples of the site-specific trends. The number of annually-averaged nutricline depths used to construct 10,000 random populations are the sample size (n) in the MToRP columns. Each *p* value in the MToRP columns was the fraction of MToRP that had a greater absolute value than the absolute value of the median trend from the observations. The sample size for the sign test was the number of unique sites. The two groups for the Kruskal-Wallis test had the same sample sizes as the sign tests.

Table S4: Regional trends from GO-SHIP

		T_{NO3}				T_{PO4}				
Region	Spatial bounds	Median (m yr^{-1})	$CI_{95\%}$ (m yr^{-1})	MToRP two-tailed *p* value	Sign test *p* value	Median (m yr^{-1})	$CI_{95\%}$ (m yr^{-1})	MToRP two-tailed *p* value	Sign test *p* value	Kruskal-Wallis test *p* value
North hemisphere	1°N to 45°N	-0.02	-0.16, 0.13	0.8 (n = 3,344)	0.8 (n = 995)	0.13	-0.05, 0.35	0.03 (n = 3,491)	0.1 (n = 982)	0.3
South hemisphere	-45°N to -1°N	-0.27	-0.40, -0.09	2×10^{-4} (n = 2,142)	1×10^{-3} (n = 849)	0.60	0.41, 0.71	0 (n = 1,738)	2×10^{-7} (n = 643)	5×10^{-11}
North Pacific	15°N to 45°N, 120°E to -100°E	-0.03	-0.26, 0.18	0.8 (n = 1,496)	0.9 (n = 422)	0.26	-0.05, 0.50	7×10^{-3} (n = 1,545)	0.1 (n = 392)	0.4
North Atlantic	15°N to 45°N, -75°E to -10°E	-0.40	-0.80, -0.04	3×10^{-3} (n = 979)	0.04 (n = 312)	0.06	-0.28, 0.60	0.8 (n = 1,069)	0.9 (n = 342)	0.05
South Pacific	-45°N to -15°N, 120°E to -70°E	-0.45	-0.75, -0.16	1×10^{-4} (n = 844)	7×10^{-4} (n = 346)	0.65	0.32, 0.86	2×10^{-4} (n = 614)	2×10^{-4} (n = 220)	3×10^{-9}
South Atlantic	-45°N to -15°N, -70°E to 20°E	0.06	-0.12, 0.31	0.6 (n = 438)	0.6 (n = 173)	0.72	0.52, 1.24	8×10^{-3} (n = 352)	3×10^{-4} (n = 130)	2×10^{-3}
Indian Ocean	-45°N to -15°N, 30°E to 115°E	-0.41	-0.80, 0.29	0.10 (n = 510)	0.2 (n = 210)	0.50	0.01, 0.93	0.02 (n = 503)	0.07 (n = 199)	0.06

The $[NO_3^-]$ and $[PO_4^{3-}]$ thresholds to define Z_{NO3} and Z_{PO4} were 3 µmol kg^{-1} and 3/16 µmol kg^{-1} respectively. Site-specific trends were determined from nutricline depths deeper than 50 m depth and from years 1972 – 2022. The 95% confidence intervals ($CI_{95\%}$) of the medians were calculated by generating 10,000 bootstrap samples of the site-specific trends. The number of annually-averaged nutricline depths used to construct 10,000 random populations are the sample size (n) in the MToRP columns. Each *p* value in the MToRP columns was the fraction of MToRP that had a greater absolute value than the absolute value of the median trend from the observations. The sample size for the sign test was the number of unique sites. The two groups for the Kruskal-Wallis test had the same sample sizes as the sign tests.

Table S5: Residual T_{PO4} vs. ocean nitrogen fixation rates in CMIP6

Scenario	Slope (m x [Tg N_{fix}]$^{-1}$)	Slope SE (m x [Tg N_{fix}]$^{-1}$)	R^2	*p* value
historical	2.5×10^{-4}	1.1×10^{-4}	0.3	0.04 (n = 15)
ssp245	1.0×10^{-3}	2.1×10^{-4}	0.7	8×10^{-4} (n = 11)
ssp585	1.3×10^{-3}	2.4×10^{-4}	0.8	4×10^{-4} (n = 11)

Statistics of the linear regressions for the residual T_{PO4} vs. ocean nitrogen fixation rates across CMIP6 models.

Table S6: CMIP6 models with scenarios and variables of interest

Model	historical<br>no3, po4, thetao & so	ssp245<br>no3, po4, thetao & so	ssp585<br>no3, po4, thetao & so	historical<br>intpn2	ssp245<br>intpn2	ssp585<br>intpn2
ACCESS-ESM1-5	x	x	x			
CESM2	x	x	x	x	x	x
CESM2-FV2	x			x		
CESM2-WACCM	x	x	x	x	x	x
CESM2-WACCM-FV2	x			x		
CMCC-ESM2	x	x	x			
CNRM-ESM2-1	x	x	x	x	x	x
EC-Earth3-CC	x	x	x	x	x	x
GFDL-ESM4	x	x	x	x	x	x
IPSL-CM5A2-INCA	x					
IPSL-CM6A-LR	x	x	x	x	x	x
IPSL-CM6A-LR-INCA	x			x		
KIOST-ESM	x	x	x			
MIROC-ES2H	x	x	x			
MIROC-ES2L	x	x	x	x	x	x
MPI-ESM-1-2-HAM	x			x		
MPI-ESM1-2-HR	x	x	x	x	x	x
MPI-ESM1-2-LR	x	x	x	x	x	x
MRI-ESM2-0	x		x			
NorESM2-LM	x	x	x	x	x	x
NorESM2-MM	x	x	x	x	x	x
UKESM1-0-LL	x	x	x			

Variables available in each model and scenario are denoted by an "x." Ocean temperature is "thetao," salinity is "so," and nitrogen fixation is "intpn2." Model variables were from the r1i1p1f1 experiment version if it was available.

SI References:

1. CCHDO Hydrographic Data Office (2023). CCHDO Hydrographic Data Archive, Version 2023-12-01. In CCHDO Hydrographic Data Archive. UC San Diego Library Digital Collections. https://doi.org/10.6075/J0CCHKOT.
2. R. M. Key *et al.* (2015). Global Ocean Data Analysis Project, Version 2 (GLODAPv2), ORNL/CDIAC-162, ND-P093. Carbon Dioxide Information Analysis Center (CDIAC) https://doi.org/10.3334/CDIAC/OTG.NDP093_GLODAPV2.
3. A. Olsen *et al.*, The Global Ocean Data Analysis Project version 2 (GLODAPv2) – an internally consistent data product for the world ocean. *Earth System Science Data* **8**, 297–323 (2016).
4. T. P. Boyer *et al.* (2018). World Ocean Database 2018. A.V. Mishonov, Technical Ed., NOAA Atlas NESDIS 87.
5. N. P. Fofonoff, R. C. Millard, Calculation of Physical Properties of Seawater. UNESCO (1991).
6. S. Becker *et al.*, GO-SHIP Repeat Hydrography Nutrient Manual: The Precise and Accurate Determination of Dissolved Inorganic Nutrients in Seawater, Using Continuous Flow Analysis Methods. *Front. Mar. Sci.* **7**, 581790 (2020).
7. L. I. Gordon, J. C. Jennings, A. A. Ross, J. M. Krest, A Suggested Protocol for Continuous Flow Automated Analysis of Seawater Nutrients (Phosphate, Nitrate, Nitrite and Silicic Acid) in the WOCE Hydrographic Program and the Joint Global Ocean Fluxes Study (1993).
8. N. P. Fofonoff, R. C. Millard, Algorithms for the computation of fundamental properties of seawater. UNESCO (1983).
9. C. C. Leroy, F. Parthiot, Depth-pressure relationships in the oceans and seas. *The Journal of the Acoustical Society of America* **103**, 1346–1352 (1998).
10. P. Cermeño *et al.*, The role of nutricline depth in regulating the ocean carbon cycle. *Proceedings of the National Academy of Sciences* **105**, 20344–20349 (2008).
11. C. A. Garcia *et al.*, Nutrient supply controls particulate elemental concentrations and ratios in the low latitude eastern Indian Ocean. *Nature Communications* **9**, 4868 (2018).
12. K. Richardson, J. Bendtsen, Vertical distribution of phytoplankton and primary production in relation to nutricline depth in the open ocean. *Marine Ecology Progress Series* **620**, 33–46 (2019).
13. C. C. James *et al.*, Influence of nutrient supply on plankton microbiome biodiversity and distribution in a coastal upwelling region. *Nature Communications* **13**, 2448 (2022).
14. D. M. Karl *et al.*, Ecological nitrogen-to-phosphorus stoichiometry at station ALOHA. *Deep Sea Research Part II: Topical Studies in Oceanography* **48**, 1529–1566 (2001).
15. M. M. Omand, A. Mahadevan, The shape of the oceanic nitracline. *Biogeosciences* **12**, 3273–3287 (2015).
16. C. de Boyer Montégut, G. Madec, A. S. Fischer, A. Lazar, D. Iudicone. Mixed layer depth over the global ocean: An examination of profile data and a profile-based climatology. *Journal of Geophysical Research: Oceans* **109**, (2004).
17. A. C. Redfield, On the proportions of organic derivatives in sea water and their relation to the composition of plankton. *Liverpool: University Press of Liverpool* (1934).
18. M. D. Patey *et al.*, Determination of nitrate and phosphate in seawater at nanomolar concentrations. *TrAC Trends in Analytical Chemistry* **27**, 169–182 (2008).
19. A. C. Martiny *et al.*, Biogeochemical controls of surface ocean phosphate. *Science Advances* **5**, eaax0341 (2019).

20. M. Dai *et al.*, Upper Ocean Biogeochemistry of the Oligotrophic North Pacific Subtropical Gyre: From Nutrient Sources to Carbon Export. *Reviews of Geophysics* **61**, e2022RG000800 (2023).
21. V. Silkin *et al.*, Phytoplankton Dynamics and Biogeochemistry of the Black Sea. *Journal of Marine Science and Engineering* 11, 1196 (2023).
22. K. Matsumoto, T. Tanioka, R. Rickaby, Linkages Between Dynamic Phytoplankton C:N:P and the Ocean Carbon Cycle Under Climate Change. *Oceanography* **33**, 44–52 (2020).
23. A. B. Kara, P. A. Rochford, H. E. Hurlburt, Mixed layer depth variability over the global ocean. *Journal of Geophysical Research: Oceans* **108**, (2003).
24. M. Aoyama *et al.*, Recent Comparability of Oceanographic Nutrients Data: Results of a 2003 Intercomparison Exercise Using Reference Materials. *Analytical Sciences* **23**, 1151–1154 (2007).
25. H. E. Garcia *et al.* (2019). World Ocean Atlas 2018. NOAA National Centers for Environmental Information.
26. S. K. Lauvset *et al.*, A new global interior ocean mapped climatology: the 1° × 1° GLODAP version 2. *Earth System Science Data* **8**, 325–340 (2016).
27. M. J. Church, M. W. Lomas, F. Muller-Karger, Sea change: Charting the course for biogeochemical ocean time-series research in a new millennium. *Deep Sea Research Part II: Topical Studies in Oceanography* **93**, 2–15 (2013).
28. R. Séférian *et al.*, Tracking Improvement in Simulated Marine Biogeochemistry Between CMIP5 and CMIP6. *Current Climate Change Reports* **6**, 95–119 (2020).
29. B. C. O'Neill *et al.*, The Scenario Model Intercomparison Project (ScenarioMIP) for CMIP6. *Geoscientific Model Development* **9**, 3461–3482 (2016).
30. M. Meinshausen *et al.*, Historical greenhouse gas concentrations for climate modelling (CMIP6). *Geoscientific Model Development* **10**, 2057–2116 (2017).
31. M. C. Long *et al.*, Simulations With the Marine Biogeochemistry Library (MARBL). *Journal of Advances in Modeling Earth Systems* **13**, e2021MS002647 (2021).
32. J. Yu *et al.* (2024). CESM2.2-8P4Z data supporting Yu et al. (2024): Simulating ecosystem dynamics and marine biogeochemical cycles with multiple plankton functional types. Zenodo https://doi.org/10.5281/zenodo.13355401.
33. N. A. Wiseman, J. K. Moore, B. S. Twining, D. S. Hamilton, N. M. Mahowald, Acclimation of Phytoplankton Fe:C Ratios Dampens the Biogeochemical Response to Varying Atmospheric Deposition of Soluble Iron. *Global Biogeochemical Cycles* **37**, e2022GB007491 (2023).
34. R. T. Letscher, J. K. Moore, A. C. Martiny, M. W. Lomas, Biodiversity and Stoichiometric Plasticity Increase Pico-Phytoplankton Contributions to Marine Net Primary Productivity and the Biological Pump. *Global Biogeochemical Cycles* **37**, e2023GB007756 (2023).
35. H. Tsujino *et al.*, JRA-55 based surface dataset for driving ocean–sea-ice models (JRA55-do). *Ocean Modelling* **130**, 79–139 (2018).
36. A. Krishnamurthy, J. K. Moore, N. Mahowald, C. Luo, C. S. Zender, Impacts of atmospheric nutrient inputs on marine biogeochemistry. *Journal of Geophysical Research: Biogeosciences* **115**, (2010).
37. E. Y. Kwon *et al.*, Nutrient uptake plasticity in phytoplankton sustains future ocean net primary production. *Science Advances* **8**, eadd2475 (2022).
38. T. J. Browning, C. M. Moore, Global analysis of ocean phytoplankton nutrient limitation reveals high prevalence of co-limitation. *Nature Communications* **14**, 5014 (2023).

39. L. J. Ustick *et al.*, Metagenomic analysis reveals global-scale patterns of ocean nutrient limitation. *Science* **372**, 287–291 (2021).
40. B. Huang *et al.* (2017). NOAA Extended Reconstructed Sea Surface Temperature (ERSST), Version 5. NOAA National Centers for Environmental Information. https://doi.org/10.7289/V5T72FNM.
41. E. J. Webb, B. I. Magi, The Ensemble Oceanic Niño Index. *International Journal of Climatology* **42**, 5321–5341 (2022).
